# HepatoDyn: A Dynamic Model of Hepatocyte Metabolism That Integrates ^13^C Isotopomer Data

**DOI:** 10.1371/journal.pcbi.1004899

**Published:** 2016-04-28

**Authors:** Carles Foguet, Silvia Marin, Vitaly A. Selivanov, Eric Fanchon, Wai-Nang Paul Lee, Joan J. Guinovart, Pedro de Atauri, Marta Cascante

**Affiliations:** 1 Department of Biochemistry and Molecular Biomedicine, Faculty of Biology, Universitat de Barcelona, Barcelona, Spain; 2 Institute of Biomedicine of Universitat de Barcelona (IBUB) and Associated Unit to CSIC, Barcelona, Spain; 3 UGA – CNRS, TIMC-IMAG UMR 5525, Grenoble, France; 4 Department of Pediatrics, Los Angeles Biomedical Research Institute, Torrance, California, United States of America; 5 Institute for Research in Biomedicine (IRB Barcelona), The Barcelona Institute of Science and Technology, Barcelona, Spain; University of California San Diego, UNITED STATES

## Abstract

The liver performs many essential metabolic functions, which can be studied using computational models of hepatocytes. Here we present HepatoDyn, a highly detailed dynamic model of hepatocyte metabolism. HepatoDyn includes a large metabolic network, highly detailed kinetic laws, and is capable of dynamically simulating the redox and energy metabolism of hepatocytes. Furthermore, the model was coupled to the module for isotopic label propagation of the software package IsoDyn, allowing HepatoDyn to integrate data derived from ^13^C based experiments. As an example of dynamical simulations applied to hepatocytes, we studied the effects of high fructose concentrations on hepatocyte metabolism by integrating data from experiments in which rat hepatocytes were incubated with 20 mM glucose supplemented with either 3 mM or 20 mM fructose. These experiments showed that glycogen accumulation was significantly lower in hepatocytes incubated with medium supplemented with 20 mM fructose than in hepatocytes incubated with medium supplemented with 3 mM fructose. Through the integration of extracellular fluxes and ^13^C enrichment measurements, HepatoDyn predicted that this phenomenon can be attributed to a depletion of cytosolic ATP and phosphate induced by high fructose concentrations in the medium.

## Author Summary

Despite the key role of hepatocytes in carbohydrate and lipid homeostasis, available dynamic models of hepatocyte metabolism tend to be limited to a single pathway and/or are based on assumptions of constant concentrations of key metabolites involved in redox and energy metabolism (ATP, NAD, NADPH etc.). Furthermore, most dynamic models are unable to integrate information from ^13^C based experiments. ^13^C based experiments allow us to infer the relative activity of alternative pathways and hence are highly useful for indicating flux distributions. To overcome these limitations, we developed HepatoDyn, a dynamic model of hepatic metabolism. HepatoDyn uses a large metabolic network including key pathways such as glycolysis, the Krebs cycle, the pentose phosphate pathway and fatty acid metabolism, and dynamically models the concentrations of metabolites involved in the redox and energy metabolism of hepatocytes. In addition, the model was coupled to the label propagation module of the package IsoDyn, allowing it to integrate data from ^13^C based experiments to assist in the parametrization process. These features make HepatoDyn a powerful tool for studying the dynamics of hepatocyte metabolism.

## Introduction

No other organ performs as many physiological functions as the liver. The liver is responsible for detoxification, bile acid and blood proteins synthesis, plays a key role in the inflammatory response and, above all, it is a key regulator of glucose and lipid homeostasis in blood. Most of its functions and properties can be linked to hepatocytes, the most abundant cell type in liver, and therefore hepatocytes are often used as a model to study liver function and pathologies [1]. Accordingly, computational modelling of hepatocyte metabolism has received a great deal of interest.

Recently, genome scale metabolic reconstructions based on stoichiometric modelling techniques have been successfully used to model hepatocyte metabolism [2–4]. However, stoichiometric models provide a static picture of metabolism based on mass balance equations and the assumption that the system is under a strict steady state. In these models each reaction step is described by only one parameter, its steady state flux [5]. The alternative is to use dynamic metabolic models, usually referred to as kinetic models. They are based on building a system of ordinary differential equations (ODEs), with kinetic laws describing transport and chemical transformations for each reaction-step and parameters describing biochemical and biophysical constraints. Kinetic modelling has two main advantages over stoichiometric based modelling; firstly, it is capable of performing dynamic simulations, that is to say, it can predict the variation in metabolite concentrations and fluxes over time outside of the steady state. Secondly, it can follow the global effects of constraints emerging from the specific kinetic properties of enzymes, post-translational modifications and regulatory circuits, thus revealing the complex regulation of the system. Over the years, multiple kinetics models of hepatocyte metabolism have been developed [6–11]. The main limitation of kinetic models is that they are complex to build and parametrize. Due to this complexity, kinetic models of hepatocyte metabolism available in the literature contain only a small number of reactions and, with some exceptions [11], are often limited to a single pathway. Furthermore, with the exception of some models focused on mitochondria [8, 9], most of them assume a constant redox and energy state, which limits their application. In fact, despite the huge interest in hepatocyte metabolism, there are no models capable of adequately modelling the effects of the energy and redox dynamics on hepatocyte core metabolism. Additionally, while ^13^C experiments have proven their usefulness in studying the metabolism of hepatocyte under metabolic steady state [12–24], there was only one kinetic model of hepatocyte capable of integrating ^13^C data [10].

In this work, we present HepatoDyn (**Hepato**cyte **Dyn**amics) a model of hepatocyte core metabolism capable of simulating the redox (NAD/NADH, NADP/NADPH, etc.) and energy (ATP/ADP/AMP, etc.) dynamics. The model includes glycolysis, gluconeogenesis, glycogen metabolism, the pentose phosphate pathway, the Krebs cycle and fatty acid metabolism as well as reactions associated with energy and redox metabolism (respiratory chain, malate/aspartate shuttle, glycerol phosphate shuttle, etc.). To our knowledge, no model of such size capable of dynamic redox and energy metabolism simulations exists in the literature. Furthermore, the model was coupled to the module for isotopic label propagation of the software package IsoDyn [25, 26]. This enables HepatoDyn to integrate data from ^13^C based experiments to assist in the parametrization process, regardless of whether experimental measurements correspond to an isotopic steady state. The latter is a key feature because the levels of isotopic label enrichment are often a non-steady phenomenon with long transition times [27]. Therefore, HepatoDyn is a very powerful tool capable of taking advantage of both the constraints derived from a detailed tissue-specific kinetic model and data derived from ^13^C based experiments to simulate hepatocytes.

In the last decades there has been a significant increase in fructose in our diets [28] and accordingly there is great interest in studying the potential effects of fructose in the metabolism [29–32]. To date, fructose-rich diets have been associated with many adverse metabolic conditions, such as nonalcoholic fatty liver disease, insulin resistance and obesity [28, 33, 34], most of which are directly or indirectly related to abnormal hepatocyte function. Therefore, we used HepatoDyn to study the short-term response of hepatocyte metabolism to different concentrations of fructose.

## Materials and Methods

### Experimental Methods

#### Materials

[1,2-^13^C_2_]D-glucose (>99% enriched) and [U-^13^C_6_]D-fructose (>99% enriched) were purchased from Isotec (Miamisburg, OH, USA), and other reagents used were from Sigma-Aldrich Company (St. Louis, MO, USA).

#### Animals

180–200 g male Wistar rats were used. They were maintained in a 12 h:12 h light-dark cycle with free access to standard laboratory rat chow pellets (Panlab) and water. Animals were deprived of food 24 h prior to hepatocyte isolation. Experiments were conducted according to guidelines accepted by the University Animal Care and Use Committee. Appropriate measures were taken to minimize pain or discomfort in the animals.

#### Preparation of cells and incubation

Suspensions of isolated parenchymal liver cells were prepared from 24-h starved animals as described previously [35]. Cell suspensions were incubated at 37°C with gassing and continuous shaking (160 strokes/min) for 2 h with Krebs–Ringer bicarbonate buffer of pH 7.4 containing glucose and fructose. At the end of the incubations, cells were centrifuged and the incubation media and cell pellets were obtained.

#### Measurement of metabolites

Glycogen content from cell pellets and glucose and lactate concentrations in incubation media were determined as described previously [19].

#### Gas chromatography/ mass spectrometry sample processing and analysis

Incubation media were processed for isolation of lactate, glucose, and glutamate using previously established methods [36, 37]. To analyse fructose isotopologues distribution, lyophilized incubation medium was treated with 0.5 N sodium borohydride in methanol for 2 h at room temperature, causing both fructose and glucose to be transformed to sorbitol. The resulting sorbitol was then isolated by ion exchange chromatography as described for glucose [37]. Glycogen was isolated from cell pellets as described previously [19]. Once isolated, glucose from the medium or from hydrolysed glycogen, as well as lactate, glutamate and sorbitol were derivatized for gas chromatography/mass spectrometry (GC/MS) analysis [36, 38, 39]. In the case of sorbitol, it was derivatized to its hexaacetate derivative according to a modification of the method described by Wolfe [40]. A mass selective detector HP 5973 equipment coupled to a gas chromatograph HP 6890 was used for all the metabolites as described elsewhere [36, 38, 39]. The GC/MS method for sorbitol analysis was the same as that for glucose analysis. Chemical ionization was used to obtain the molecular ion (C1-C6) of the glycogen or medium glucose molecules at m/z 328, and the same for the lactate molecule (C1-C3) at m/z 328 and sorbitol molecule (C1-C6) at m/z 375. Electron impact ionization was used to characterize the isotopologue fractions of C1-C4 (m/z 242) and C3-C6 (m/z 187) glycogen glucose fragments, as well as C2-C4 (m/z 152) and C2-C5 (m/z 198) glutamate fragments.

Spectral data were corrected using regression analysis to extract natural ^13^C enrichment from results [41]. Measurement of ^13^C label distribution determined the different relative distribution percentages of the isotopologues, m0 (without any ^13^C labels), m1 (with one ^13^C), m2 (with two ^13^C), etc.

### Building the model

A metabolic network, including those pathways deemed necessary to accurately and dynamically simulate the core metabolism of rat hepatocytes in the study conditions, was constructed based on pathways that have been reported in the literature to be active in hepatocytes [42, 43].

Each reaction in the metabolic network was assigned a kinetic law. Kinetic laws describe the dependence of each reaction flux on metabolite concentrations. They take into account the affinity of substrates and products, the reaction mechanism and the effect of activators and inhibitors on reaction fluxes. The kinetic laws used were mostly derived from existing kinetic laws described in the literature [6, 11, 44]. The exceptions were the kinetic laws for aldolase activity, which catalyses eight related elementary reactions, which were built as described in the Supplementary Material (S1 Text).

Kinetic laws are integrated with the metabolic network topology, described by the stoichiometric matrix (N), to build a system of ordinary differential equations (ODEs) that predict the evolution of metabolite concentrations, and by extension the evolution of reaction fluxes, over time. Because fluxes are provided in units of mmol *per* cell *per* minute, but ODEs are solved in units of mmol *per* litre *per* minute, in order to build the ODEs, the cell number and the volume of the compartment at which each metabolite is located must also be taken into account. Therefore, the system of ODEs can be written as:

$$\;\;\;\frac{dc\left[t\right]}{dt}=N\cdot j\left(c\left[t\right],p\right)\cdot \frac{ncell}{vol}$$ (1)

Where *j* is a vector of reaction fluxes, which is a function of the vector of metabolite concentrations (c[t]) in mM, and a vector model parameter (*p*) as defined by the kinetic laws used in the model, *ncell* is the cell number and *vol* is a vector containing the volumes of the compartment at which each metabolite is localized in litres.

In reversible reactions, forward and reverse reaction rates are computed separately with different kinetic laws, albeit sharing most of the parameters. Additionally, the fluxes of invisible reactions, that is to say, reactions that can propagate labelled carbons even though they do not change the overall concentrations of metabolites, are also computed [10]. This is necessary in order to fully simulate the propagation of ^13^C.

**Fig 1:**
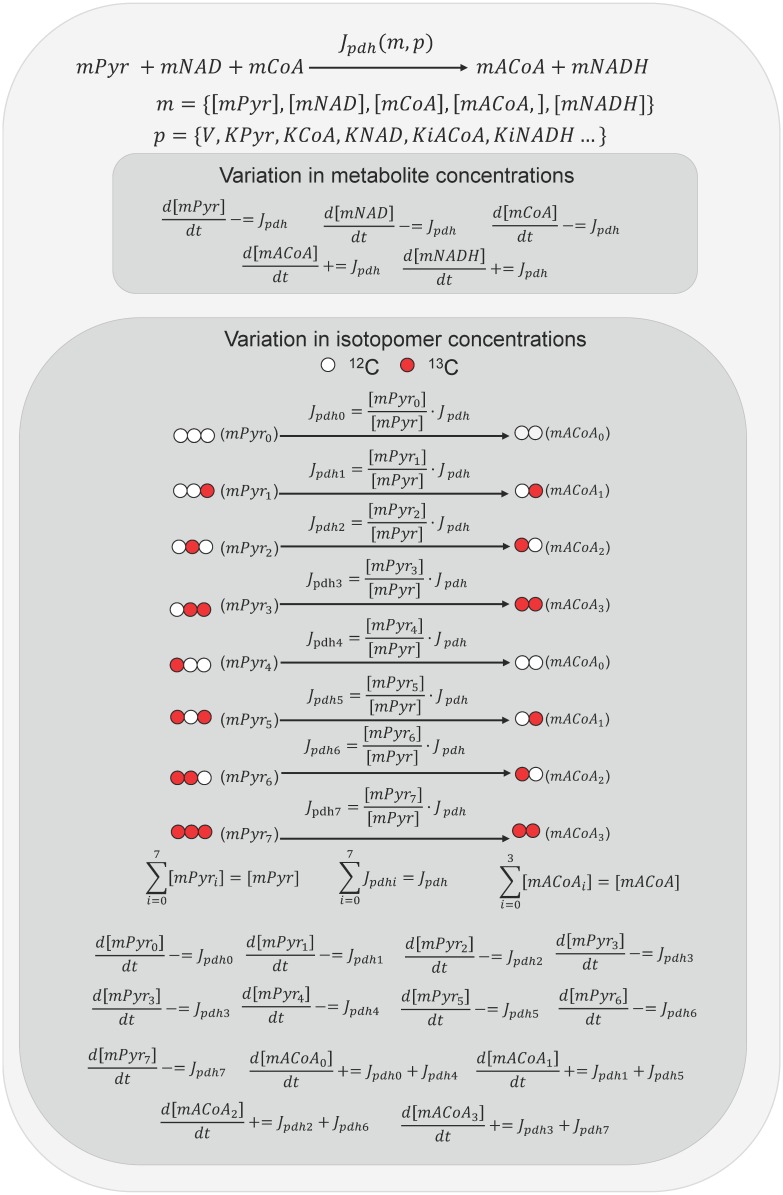
Example of how ODEs are automatically built for isotopomers and metabolites consumed or produced by the pyruvate dehydrogenase catalysed reaction (PDH). PDH irreversibly transforms mitochondrial pyruvate (mPyr), NAD (mNAD), and coenzyme A (mCoA) into mitochondrial acetyl-CoA (mACoA) and NADH (mNADH). The system of differential equations is solved taking into account all equations for total concentrations of metabolites and for concentrations of isotopomers. From the previous step in the simulation, the PDH flux (J_pdh_) is computed, which is a function of the concentrations of the reactants and products (m) and the kinetic parameters of PDH (p). For the ODEs describing the concentration of metabolites the computed value is added (+ =) and subtracted (- =) for products and substrates, respectively. For the ODE describing a particular isotopomer, the flux value is scaled according to the relative abundance of the isotopomer for the substrate (mPyr_i_) and the resulting scaled flux (J_PDHi_) is added (+ =) and subtracted (- =) to d[mACoA_i_]/dt and d[mPyr_i_]/dt, respectively. Isotopomers are not simulated for CoA, NAD or NADH because it is assumed that ^13^C from labelled substrates does not propagate to such metabolites.

To simulate the propagation of ^13^C through the metabolic network, fluxes are decomposed into isotopomer fluxes. Then, an ODE system is built using the algorithms from IsoDyn [25, 26]. The resulting ODE accounts for concentrations of all isotopomers, isomers with ^13^C substitution in specific carbon positions [24]. To avoid unnecessary complexity, isotopomers are not simulated for those metabolites where, according to the defined metabolic network, ^13^C from labelled substrates cannot be propagated. The process is briefly summarized in Fig 1.

The system of differential equations for metabolite and isotopomer concentrations is solved to predict metabolic fluxes, metabolic concentrations and isotopomer concentrations from the initial time to the defined end time.

Model predictions are for isotopomers but experimental measurements refer to isotopologues (or mass isotopomers), isomers with a specific number of ^13^C substitutions [24]. Thus, the resulting concentrations of isotopomers are converted into fractions of isotopologues, by adding up all isotopomers that correspond to each isotopologue and dividing by the total concentration of each metabolite (S1 Fig). The fractions of such isotopologues can then be compared with the experimental measurements obtained with GC coupled to MS.

### Parameterization

Kinetic parameters representing enzyme activity (*V*_*max*_ or equivalent) were fitted to the experimental data. For this process *V*_*max*_ from the reverse reaction rate in reversible reactions are assumed to be a function of the *V*_*max*_ of the forward reaction and of the equilibrium constant as described by the Haldane relationship [44]. To further reduce the number of parameters fitted, enzyme activities catalysing sequential reactions with no ramifications (the so called reactions chains) were fitted as a group. This is because in reactions chains the flux through the whole chain could be determined by any of the enzyme activities involved and consequently most of the activities of enzymes constituting the chain would be unidentifiable. Furthermore, other activities known to be unidentifiable are not fitted, such as the activities of reactions that are known to operate in rapid equilibrium in physiological conditions (glucose phosphate isomerase, triose phosphate isomerase, enolase, etc.). The remaining parameters of the kinetic model were assigned based on an extensive literature search, completed with data from Brenda [45] and UniProt [46] databases.

The fitting algorithm, a variant of the basic simulated annealing algorithm [47], seeks the set of *m* parameters (E_z_) that minimizes the objective function. The objective function (*X*^*2*^) is the square deviation between the *n* experimentally measured values (Y_*i*_) and simulated values (*Z*_*i*_) for both isotopologue fractions and total metabolite concentrations, normalized by the experimental standard deviation (σ_i_). To prevent a bias generated by very low standard deviations, a minimum threshold of 0.01 was used. Additionally, parameter sets where any metabolite reached concentrations greater than 50 mM were discarded.

$$X^{2}=\sum_{i=1}^{n}{\left(\frac{Y_{i}-Z_{i}\left(E_{1},E_{2},\ldots ,E_{m}\right)}{\sigma_{i}}\right)}^{2}$$ (2)

Consequently, the fitting algorithm seeks the set of enzyme activities that minimize the difference between experimentally measured and simulated isotopologue fractions and metabolite concentrations in the experimental conditions considered.

### Identifiability analysis

The fitting procedure provides one set of fitted parameters, which minimizes the objective function, and is referred to as the best fit parameter set. However, other sets of parameter values might result in similar or equal objective function values and are therefore as valid as the best fit. The range of acceptable variation in parameters was evaluated through an identifiability analysis. Identifiability is a property that indicates whether unknown model parameters can be determined from the available experimental data. It depends both on the structure of the model and the quality and amount of experimental data. A parameter is defined as identifiable if the confidence interval for its estimated value at a given significance level is finite [48, 49].

If we define *X*^*2*^*(*θ_i_*)* as the optimized square deviation if parameter *i* is fixed to a value of θ_i_ and the remaining parameters being fitted θ_j_ are readjusted to minimize the square deviation

$$X^{2}\left(\theta_{i}\right)={\text{min}}_{\theta_{j\neq i}}\left[x^{2}\left(\theta_{j}\right)\right]$$ (3)

then if experimental errors are assumed to follow a normal distribution, for a parameter *i*, the confidence interval can be defined as:

$$\left\{\theta_{i}|X^{2}\left(\theta_{i}\right)-X_{bf}^{2}<\Delta \alpha \right\}\;with\;\Delta \alpha =X^{2}\left(\alpha ,1\right)$$ (4)

where *X*^*2*^_*b*f_ is the best fit square deviation (optimized with no fixed parameters) and *Δα* is the significance threshold associated with a given significance level (α) with a Chi Square distribution with one degree of freedom. Accordingly, the upper and lower limit of the confidence intervals for a given parameter are estimated by respectively increasing and decreasing the value of the parameter until the square deviation difference obtained when optimizing the remaining parameters exceeds the threshold (Δα) [48].

Additionally, intervals for system dependent variables (fluxes, metabolite concentrations and isotopologue fractions at different time points) are estimated from the maximum and minimum parameter values of confidence intervals generated during the identifiability analysis.

## Results

### HepatoDyn: A kinetic model capable of integrating ^13^C based measurements

**Fig 2:**
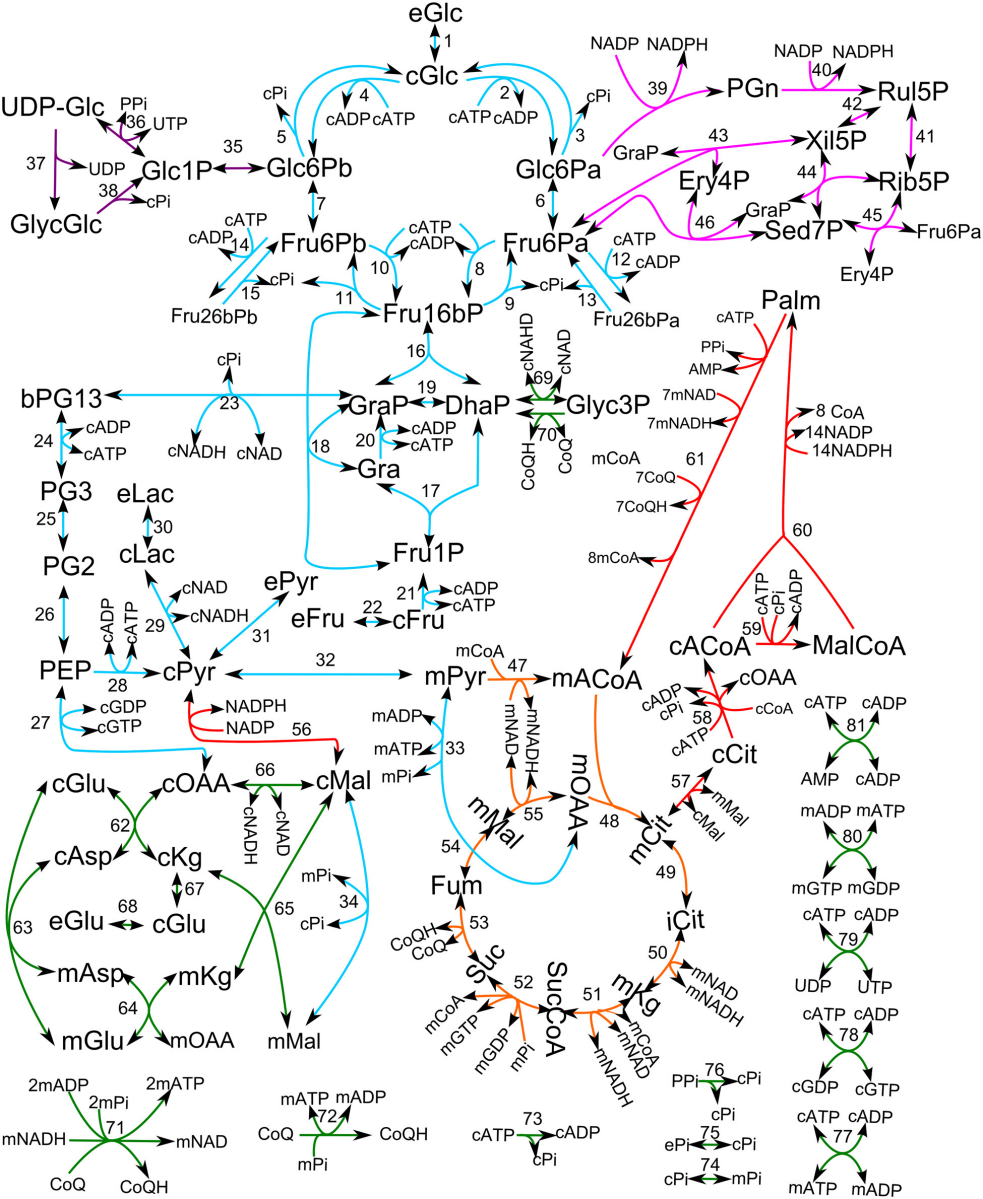
Schematic representation of the metabolic network used in the model. In this representation, reactions associated with the glycolytic and gluconeogenic pathways are coloured in blue, reactions associated with glycogen metabolism are coloured in purple, reactions associated with the pentose phosphate pathway are coloured in pink, reactions associated with the Krebs cycle are coloured in orange, reactions associated with fatty acid metabolism are coloured in red and other reactions associated with redox and energy metabolism are coloured in green. Specifically, the reactions id of each reaction represented are 1:glctr, 2: gka, 3 g6pasea, 4: gkb, 5: g6paseb, 6: gpia, 7: gpib, 8: pfkla1, 9: fbasea1, 10: pfklb1, 11: fbaseb1, 12: pfkla2, 13: fbasea2, 14: pfklb2, 15: fbaseb2, 16: aldo1, 17: aldo2, 18: aldo3, 19: tim, 20: trik, 21: fruhk, 22: frutr, 23: gapdh, 24: pgk, 25: pgm, 26: eno, 27: pepck, 28: pk, 29: ldh, 30: lactr, 31: pyrtr, 32: mpyrtr, 33: pc, 34: dic, 35: pglm, 36: ugt, 37: gs, 38: gp, 39: g6pdh, 40: pgndh, 41: rpi, 42: rul5pepi, 43: tk1, 44: tk2, 45: tk3, 46: ta, 47: pdh, 48: cs, 49: aco, 50: idh, 51: kdh, 52: scs, 53: sdh, 54: fh, 55: mmdh, 56: malic, 57: citmtr, 58: citly, 59: acoacar, 60: fasyn, 61: box, 62: aatc, 63: aspglumtrans, 64: aatm, 65: malkgmtrans, 66: cmdh, 67: transa, 68: glutr, 69: glyc3pcdh, 70: glyc3pmdh, 71: nadhdh, 72: coqhoxi, 73: atpase, 74: pimtr, 75: pitr, 76: ppase, 77: atpmtrans, 78: cndk1, 79: cndk2, 80: mndk and 81 adk. Invisible reactions are not shown for clarity. The full lists of metabolites and reactions can be found on S1 and S2 Tables respectively.

We present HepatoDyn, the first detailed model of hepatocyte core metabolism capable of dynamically simulating energy and redox metabolism. It consists of 88 reactions and 81 metabolites distributed into three compartments (extracellular, cytosolic and mitochondrial). A schematic representation of the model can be found in Fig 2 and a complete list of metabolites, reactions and compartments can be found in S1, S2 and S3 Tables, respectively.

Each reaction has an associated kinetic law and the model has a total of 470 parameters associated to kinetic laws (S4 Table). 55 of these parameters correspond to enzyme activities that were fitted to experimental data, taking parameter groups (S5 Table) into account this results in 29 independent parameters that were fitted to experimental data. To the greatest extent possible, the kinetic laws and their parameters were specific to the enzyme isoforms active in the liver.

It is worth noting, that while most of the reactions included in HepatoDyn are also present in genome scale reconstructions of hepatocyte metabolism [2–4], HepatoDyn includes complete kinetic laws and regulatory loops, which allow for dynamic and regulatory studies. Nevertheless, HepatoDyn also has 2 reactions that are absent in genome scale reconstructions of hepatocyte. Specifically, the reactions aldolase 3 (Fru16bP + Gra ↔ Fru1P + GraP) and transketolase 3 (Fru6Pa + Rib5P ↔ E4P + Sed7P). Those reactions emerge because the enzymes aldolase and transketolase allow multiple combinations of substrates and products. Additionally, HepatoDyn also incorporates the channelling of hexose phosphates to glycogen in the form of two separate pools of hexose phosphates, *a* and *b*, as previously described in the literature [10].

The kinetic model, fully parametrized, can be found in SBML format in the Supplementary Material (S1 XML and S2 XML).

In addition, HepatoDyn is capable of simulating the propagation of ^13^C from isotopically labelled substrates to metabolic intermediaries and products. This allows HepatoDyn to integrate isotopologue enrichment measurements from ^13^C based experiments greatly enhancing the predictive capabilities of the model.

HepatoDyn is provided in the Supplementary Material as a C++ program (S1 Software**)**.

### Analysing the effects of fructose on hepatocyte metabolism using HepatoDyn

The liver has a high capacity to metabolize fructose, it is estimated that up to 50% of fructose ingested is metabolized by hepatocytes [50]. Fructose metabolism in hepatocytes consists of phosphorylation of fructose to fructose 1-phosphate by fructokinase and the split of this metabolite by the liver aldolase isoform (aldolase B) into dihydroxyacetone-phosphate and glyceraldehyde, with the latter metabolite being phosphorylated by triokinase into glyceraldehyde 3-phosphate. Because fructose enters at the level of triose phosphate, bypassing the highly regulated glucokinase and phosphofructokinase steps of glycolysis, fructose uptake is largely unregulated. Consequently, the limiting step in fructose metabolism is assumed to be fructose uptake by hepatocytes, which is heavily dependent on the extracellular concentration of fructose due to the low affinity of the proteins mediating fructose transport into hepatocytes, GLUT2 and other carriers like GLUT8 [51–53].

**Fig 3:**
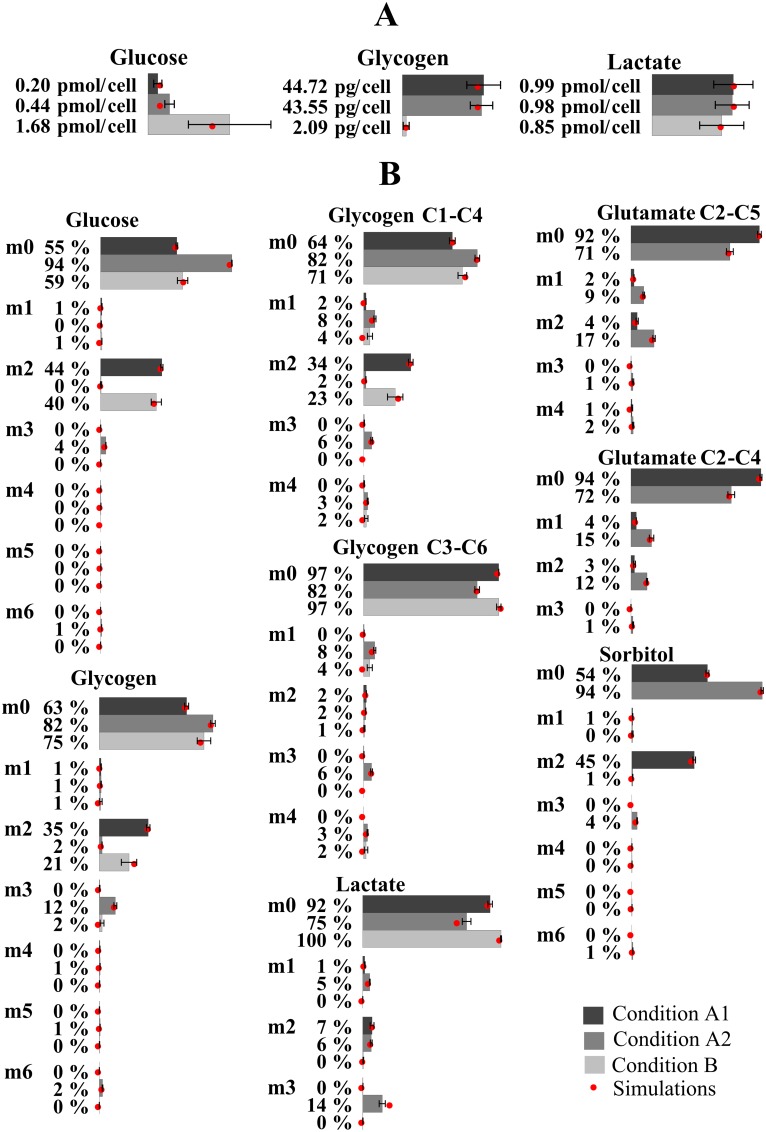
Bar graphs representing the experimentally determined metabolite productions (3.A) and isotopologue fractions (3.B) in experimental conditions. Measurements were taken after incubating hepatocytes for 2 hours with 20 mM glucose 50% enriched in [1,2-^13^C_2_]-glucose and 3 mM fructose (condition A1), 20 mM glucose and 3 mM fructose 50% enriched in [U-^13^C_6_]-fructose (condition A2) and 20 mM glucose 50% enriched in [1,2-^13^C_2_]-glucose and 20 mM fructose (condition B). The red dot indicates the value fractions simulated by HepatoDyn using the best fit parameter set. Results of the isotopologue fractions are reported as m0, m1, m2, etc. where m0, m1, m2… indicate the number of ^13^C atoms in the isotopologue fractions of a given metabolite.

As a proof of concept of the capabilities of HepatoDyn, we applied it to study the short term response of hepatocytes to incubation with 20mM glucose supplemented by either 3mM fructose or 20mM fructose. These concentrations were chosen because our experimental data showed that hepatocytes responded quite differently to them. While incubation with 20mM glucose supplemented with 3mM fructose resulted on a rapid glycogen accumulation, incubation with 20mM glucose supplemented with 20mM fructose resulted on almost no glycogen accumulation (Fig 3.A). While it has been reported that supplementation with low concentrations of fructose favours glycogen accumulation [19, 29, 54], the fact that supplementation with high fructose concentrations inhibits glycogen accumulation was not known. Furthermore, isotopologue analysis indicated that in the second condition, unlike the first condition, almost no ^13^C from labelled glucose was propagated to lactate (Fig 3.B). In both conditions lactate and glucose were produced from fructose at a similar rate. Hence it was an interesting case of study.

Specifically, HepatoDyn was used to integrate experimental measurements derived from rat hepatocytes incubated for 2 h with the following media: 20 mM glucose 50% enriched in [1,2-^13^C_2_]-glucose and 3 mM fructose (condition A1), 20 mM glucose and 3 mM fructose 50% enriched in [U-^13^C_6_]-fructose (condition A2) and 20 mM glucose 50% enriched in [1,2-^13^C_2_]-glucose and 20 mM fructose (condition B). The experimental data for condition A1 had been published previously [19]. This integration was achieved using the experimental measurements of extracellular concentrations and isotopologue fractions as input to fit the 29 independent parameters associated to enzyme activities in the model assuming that the enzyme activities, normalized by cell number (S2 Fig), were equivalent in the three conditions. Consequently, the fitting algorithm identifies a single set of parameters that allows reproduction of the three experimental conditions. It is worth noting that because conditions A1 and A2 only differ in the labelling pattern of substrates, the predicted fluxes and concentrations values will be the same in both conditions. The resulting values of the fitted parameters can be found in S6 Table. The resulting metabolites concentrations for condition A1/A2 and condition B can be found on S3 and S4 Figs respectively. The resulting fluxes for condition A1/A2 and condition B can be found on S5 and S6 Figs respectively. The resulting isotopologue fractions for key metabolites in condition A1, A2 and B can be found on S7, S8 and S9 Figs respectively. A comparison between the experimentally measured metabolite concentrations and isotopologue fractions and those simulated by the model with the best fit parameter set can be found in Fig 3.

**Fig 4:**
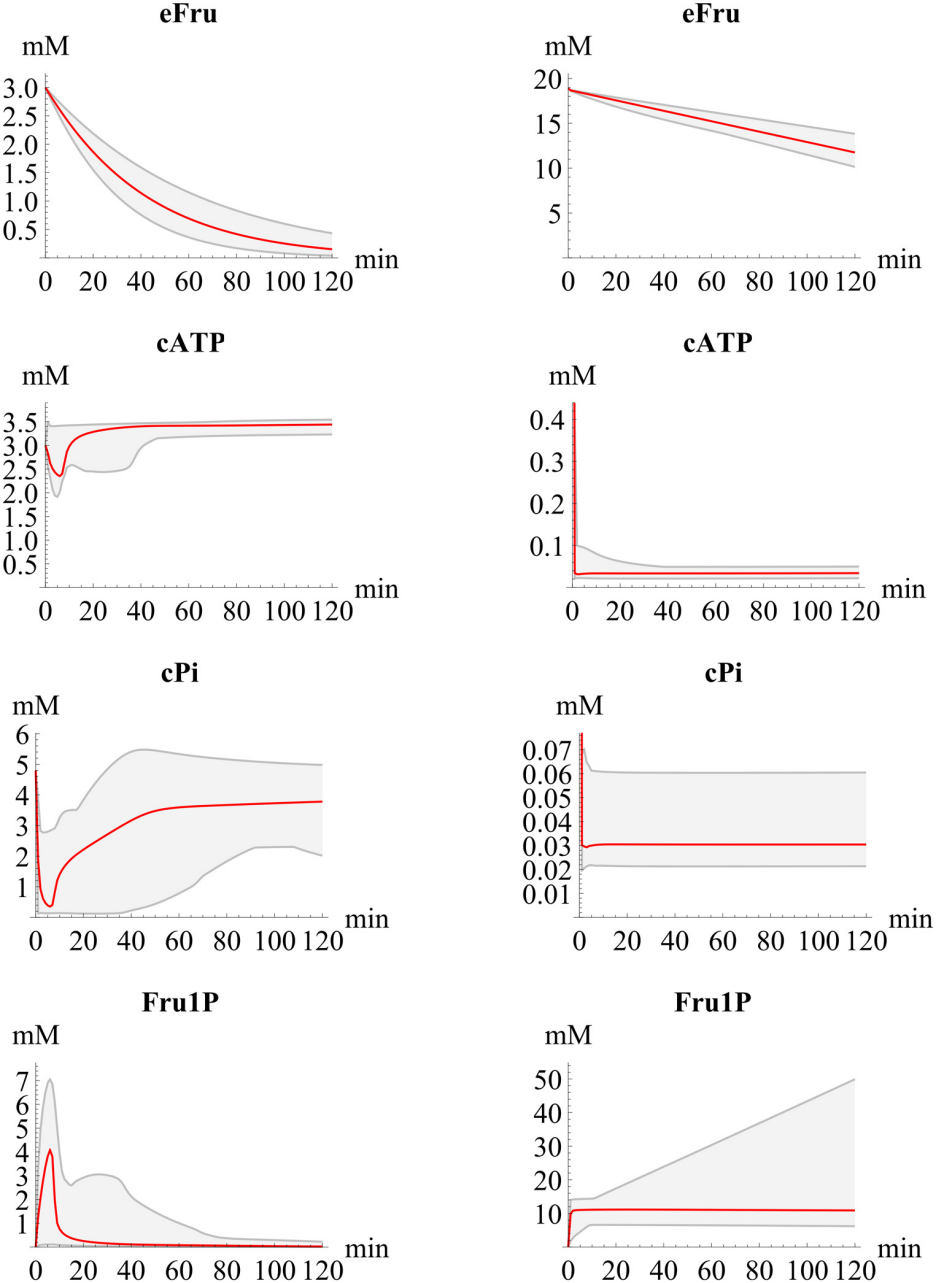
Plot of the simulated concentrations over time for extracellular fructose (eFru), fructose 1-phosphate (Fru1P), cytosolic phosphate (cPi) and cytosolic ATP (cATP). Specifically, the simulated concentrations in hepatocytes incubated with 20 mM glucose and 3 mM fructose (conditions A1 and A2, described in the main text) or 20 mM glucose and 20 mM fructose (condition B, described in the main text) are shown. The red plot indicates the values predicted with the best fit parameter set and the grey area indicates the estimated range of variations taking parameter sets within the 95% confidence intervals derived from the identifiability analysis.

High concentrations of fructose have been shown *in vivo* and *in vitro* to result in the depletion of ATP and phosphate in hepatocytes [52, 55]. This occurs due to an accumulation of fructose 1-phosphate caused by the elevated fructokinase activity [52, 55]. This phenomenon was predicted by HepatoDyn. The model predicted that a persistent cytosolic ATP and phosphate depletion would occur with an extracellular concentration of 20 mM fructose (Fig 4). This is mainly caused by an accumulation of fructose 1-phosphate, although the depletion can also be partially attributed to the accumulation of some other phosphorylated metabolites. In this context, the low glycogen synthesis observed at 20mM glucose supplemented with 20 mM fructose can be attributed to the depletion of cytosolic ATP and phosphate. Likewise, the almost non-existent propagation of ^13^C from glucose to lactate under this condition can mainly be attributed to the low glucokinase and phosphofructokinase activities caused by ATP depletion. Conversely, at 20mM glucose supplemented with 3 mM fructose, a persistent accumulation of fructose 1-phosphate does not occur. Accordingly, under this condition, ATP and phosphate are not persistently depleted (Fig 4).

### Identifiability

Overall, 25 of the 29 independent parameters were identifiable with at least 95% confidence. This remarkable degree of identifiability can be attributed to the numerous feedback regulations through the redox and energy balances (ATP/ADP, NADH/NAD, etc.), the use ^13^C data and the integration of data from multiple metabolic conditions.

Concerning the non-identifiable parameters, the non-identifiability of the aldolase activity and the activities involved in the lactate production and malate aspartate shuttle reaction chains can be attributed to the fact that the reactions associated to those pathways are predicted to be close to the equilibrium in experimental conditions, hence the system is fairly insensitive to the value of the enzyme activities associated to them. On the other hand, the non-identifiability of the citrate synthase activity arises because in our model the flux through the citrate synthase reaction can depend solely on the two activities upstream, pyruvate dehydrogenase and β-oxidation, which catalyse the production of acetyl-CoA, the substrate of citrate synthase.

Compared to parameters, fluxes and to a lesser extent concentrations, show a much narrower range of variation (S3, S4, S5 and S6 Figs). This can serve as an indication of robustness, the capacity of the system to maintain its functional properties in the face of external and internal perturbations and uncertainty [56].

Interestingly, fluxes associated with the pentose phosphate pathway and fatty acid synthesis have fairly low upper bounds in both conditions (incubation with 3 mM fructose and 20 mM glucose and incubation with 20 mM fructose and 20 mM glucose). This is consistent with hepatocytes extracted from fasted rats, as they can be expected to have low activity in fatty acid synthesis, and thus only need to generate a small amount of reductive potential (NADPH) to maintain cell functions. However, with longer incubation times, an increase in the fatty acid synthesis and pentose phosphate pathway activities and fluxes should be observed as fructose is known to increase the expression of key lipogenic enzymes in hepatocytes[28, 57, 58].

It is also worth noting that the identifiability analysis further reinforces the notion that hexose phosphate metabolism in hepatocytes is compartmentalized into two different pools as previously reported [10]. This is because most of enzyme activities present in both hexose pools have a lower bound above 0 in the confidence interval, suggesting that the separation of hexose phosphates into two separate pools must be taken into account to adequately simulate the experimental conditions. If there was no compartmentalization, all activities present in both pools would have a lower bound of 0 because they would be made redundant by the activities in the other pool.

## Discussion

Metabolic modelling is based on applying constraints to limit the space of feasible solutions for system variables, such as reaction fluxes and metabolite concentrations. Constraints can arise from different components of the model including reaction stoichiometry and kinetic laws, and from the experimental measurements integrated by the model. Consequently, the use of a highly complete metabolic network, including the fundamental balances affecting redox and energy metabolism (ATP/ADP, NAD/NADH, etc.), serve as an important set of constraints. Furthermore, the inclusion of highly detailed kinetic laws and parameters derived from the literature further constrains the solution space. For instance, important constraints that emerge from kinetic laws are regulatory circuits, such as fructose 6-phosphate inhibiting glucokinase or fructose-1-phosphate disrupting such inhibition [59–61]. Other important constraints that emerge from the kinetic laws are thermodynamics constraints, which are in the form of equilibrium constants. Finally, integrating ^13^C based data provides additional constraints such as labelling enrichments which provide information on ratios among fluxes through alternative metabolic pathways. While numerous kinetic models of hepatocytes exist in the literature [6–11], HepatoDyn is the first that is capable of integrating all the aforementioned constraints in a single model.

As a proof of concept of the capabilities of the model, we applied HepatoDyn to study the metabolic effects of high fructose concentrations on rat hepatocytes. Experimental data showed that hepatocytes behaved quite differently depending on whether they were incubated with 20mM Glucose supplemented with either 3 mM fructose or 20 mM fructose. Using HepatoDyn, we managed to find a physiological explanation for this behaviour, which involved the rapid and persistent depletion of cytosolic ATP and phosphate at 20 mM fructose, which was in accordance with information reported in the literature [52, 55]. This phenomenon has a strong dynamic component, is dependent on the kinetic properties of enzymes and on the balances involved in energy metabolism. Additionally, it may be relevant for understanding the potential adverse effects of fructose-rich diets. This is because ATP depletion impairs protein synthesis and induces inflammatory and prooxidative changes and thus, in a fructose-rich diet, this depletion might result in increased susceptibility of hepatocytes to injury leading to adverse hepatic conditions such as nonalcoholic fatty liver disease [62].

Furthermore, HepatoDyn has countless applications that go beyond studying the effects of fructose. For instance, HepatoDyn can be used to study liver centric metabolic diseases such as diabetes. Given that HepatoDyn is capable of dynamically simulating the redox and energetic state of hepatocytes, it can be used to better understand the mechanism of action of anti-diabetic drugs like metformin which target the energetic and redox metabolism [63] as well as identifying new drug targets. HepatoDyn can also be used to study the relative contribution of different reactions to redox and energy balances in different conditions. Therefore, potential applications of HepatoDyn can be to analyse the ATP consumption or production associated to different pathways or the relative contribution of the glycerol phosphate shuttle and the malate aspartate shuttle to the transfer of reducing equivalents between the cytosol and the mitochondrial matrix. Last, but not least, new reactions can easily be added to HepatoDyn provided kinetic mechanisms and kinetic information such as affinity constants or inhibition constants are known for the enzymes catalysing those reactions. Likewise, through the modification of reactions and kinetic laws specific to hepatocytes, HepatoDyn can be adapted to other cell types.

## Supporting Information

S1 Fig Example of how concentrations of pyruvate’s isotopomers ([Pyr_i_]) are converted to isotopologue fractions (fPyr_mz_). This is achieved by adding up all isotopomers that correspond to each isotopologue of pyruvate and dividing by the total concentration of pyruvate.
(TIFF)

S2 Fig Bar graph representing the concentration of cells in each experimental condition described in the main text (A1, A2 and B). (TIFF)

S3 Fig Plots of the simulated concentrations over time for hepatocytes incubated with 3 mM fructose and 20 mM glucose (conditions A1 and A2, described in the main text). The red plot indicates the values predicted with the best fit parameter set and the grey area indicates the estimated range of variations taking parameter sets within the 95% confidence intervals derived from the identifiability analysis.
(TIFF)

S4 Fig Plots of the simulated concentrations over time for hepatocytes incubated with 20 mM fructose and 20 mM glucose (condition B, described in the main text). The red plot indicates the values predicted with the best fit parameter set and the grey area indicates the estimated range of variations taking parameter sets within the 95% confidence intervals derived from the identifiability analysis.
(TIFF)

S5 Fig Plots of the simulated fluxes over time for hepatocytes incubated with 3 mM fructose and 20 mM glucose (conditions A1 and A2, described in the main text). The red plot indicates the values predicted with the best fit parameter set and the grey area indicates the estimated range of variations taking parameter sets within the 95% confidence intervals derived from the identifiability analysis.
(TIFF)

S6 Fig Plots of the simulated fluxes over time for hepatocytes incubated with 20 mM fructose and 20 mM glucose (condition B, described in the main text). The red plot indicates the values predicted with the best fit parameter set and the grey area indicates the estimated range of variations taking parameter sets within the 95% confidence intervals derived from the identifiability analysis.
(TIFF)

S7 Fig Plots of the simulated isotopologue fractions over time for Glucose, Glycogen, Sorbitol, Lactate and Glutamate for hepatocytes incubated with 20 mM glucose 50% enriched in [1,2-^13^C_2_]-glucose and 3 mM fructose (condition A1, described in the main text). The isotopologue fractions of sorbitol refer to sorbitol derived from simulated Glucose and simulated Fructose. The red plot indicates the values predicted with the best fit parameter set and the grey area indicates the estimated range of variations taking parameter sets within the 95% confidence intervals derived from the identifiability analysis. Isotopologue fractions are reported as m0, m1, m2, etc. where m0, m1, m2… indicate the number of ^13^C atoms in the fraction.
(TIFF)

S8 Fig Plots of the simulated isotopologue fractions over time for Glucose, Glycogen, Sorbitol, Lactate and Glutamate for hepatocytes incubated with 20 mM glucose and 3 mM fructose 50% enriched in [U-^13^C_6_]-fructose (condition A2, described in the main text). The isotopologue fractions of sorbitol refer to sorbitol derived from simulated Glucose and simulated Fructose. The red plot indicates the values predicted with the best fit parameter set and the grey area indicates the estimated range of variations taking parameter sets within the 95% confidence intervals derived from the identifiability analysis. Isotopologue fractions are reported as m0, m1, m2, etc. where m0, m1, m2… indicate the number of ^13^C atoms in the fraction.
(TIFF)

S9 Fig Plots of the simulated isotopologue fractions over time for Glucose, Glycogen, Sorbitol, Lactate and Glutamate for hepatocytes incubated with 20 mM glucose 50% enriched in [1,2-^13^C_2_]-glucose and 20 mM fructose (condition B, described in the main text). The isotopologue fractions of sorbitol refer to sorbitol derived from simulated Glucose and simulated Fructose. The red plot indicates the values predicted with the best fit parameter set and the grey area indicates the estimated range of variations taking parameter sets within the 95% confidence intervals derived from the identifiability analysis. Isotopologue fractions are reported as m0, m1, m2, etc. where m0, m1, m2… indicate the number of ^13^C atoms in the fraction.
(TIFF)

S1 Table Metabolites included in the model. This table describes all metabolites included in the model and provides abbreviation, full name, initial concentrations used as initial values for simulations and whether they are dependent variables or assumed as constants. For initial concentrations, “A1, “A2” and “B” superscripts refer to values specific for conditions A1 & A2 and B, respectively, as described in the main text.
(PDF)

S2 Table Reactions included in the model. This table describes all the reactions included in the metabolic network used in the model and indicates abbreviation, full name and stoichiometry. In reaction stoichiometry, ↔ denotes reversible reactions and → denotes irreversible reactions.
(PDF)

S3 Table Compartments included in the model. This table describes the three compartments included in the model and their volumes.
(PDF)

S4 Table Parameters of the kinetic model. This table describes all the parameters of the kinetic model sorted by the reaction to which they are associated.
(PDF)

S5 Table Parameter groups. This table describes the parameters representing enzyme activities that are fitted as a group, indicating the relative value of the enzyme activities associated to each group.
(PDF)

S6 Table Parameters fitted to experimental conditions. This table describes the predicted values for all parameters that have been fitted to the experimental data. In addition to the best fit value, the confidence intervals for 95% confidence according to identifiability analysis are also shown.
(PDF)

S1 Text Kinetic laws used for the aldolase reaction. This text describes the kinetic laws used for the aldolase reactions and how they have been constructed.
(PDF)

S1 XML Kinetic model 3mM Fructose. SBML version of the kinetic model described in Methods with experiment specific variables set to match those of the experiment in which hepatocytes were incubated with 20 mM glucose and 3 mM fructose (conditions A1 and A2 described in the main text).
(XML)

S2 XML Kinetic model 20mM Fructose. SBML version of the kinetic model described in Methods with experiment specific variables set to match those of the experiment in which hepatocytes were incubated with 20 mM glucose and 20 mM fructose (condition B described in the main text).
(XML)

S1 Software HepatoDyn. The software HepatoDyn (that integrates both the kinetic model and the label propagation model) and its source code. Further information about the software is provided in the readme file packaged with the software.
(ZIP)
