# Supplementary figures and images for: HepatoDyn: A Dynamic Model of Hepatocyte Metabolism That Integrates 13C Isotopomer Data
Source: PLoS Comput Biol. 2016 Apr 28;12(4):e1004899. doi: 10.1371/journal.pcbi.1004899 (PMC4849781; doi:10.1371/journal.pcbi.1004899)

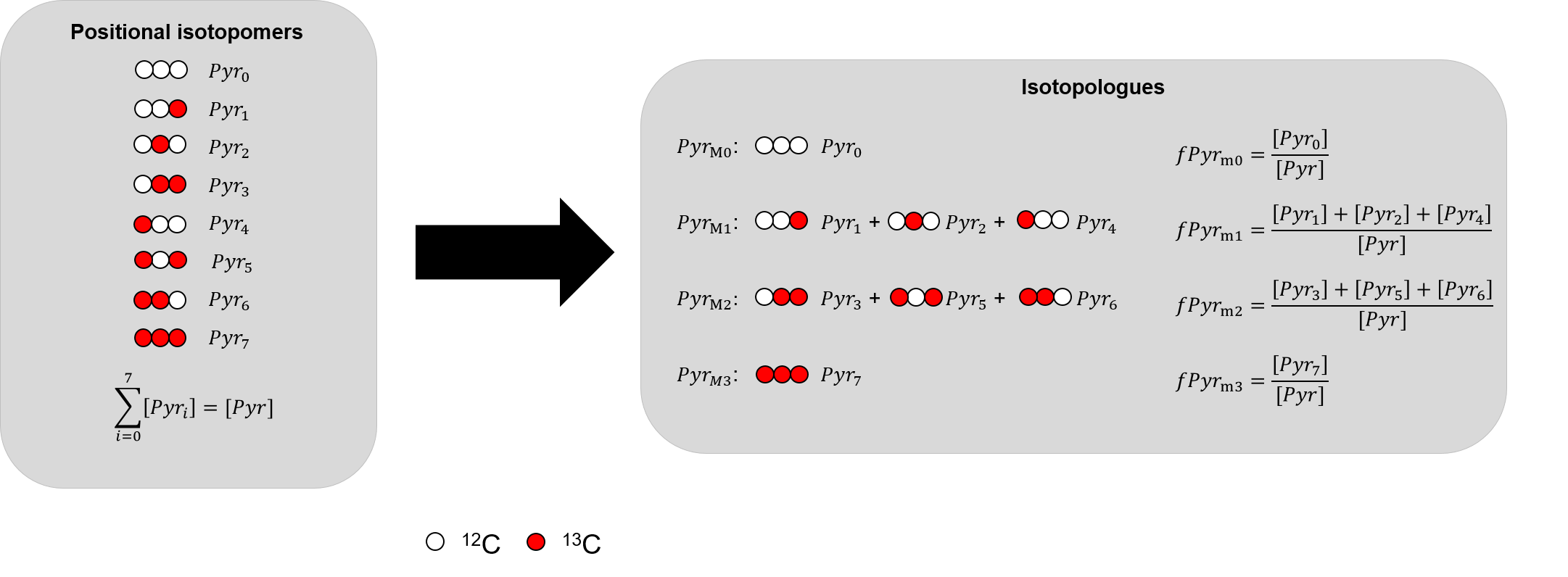

Supplement: S1 Fig — This is achieved by adding up all isotopomers that correspond to each isotopologue of pyruvate and dividing by the total concentration of pyruvate. (TIFF) [file pcbi.1004899.s001.tiff]

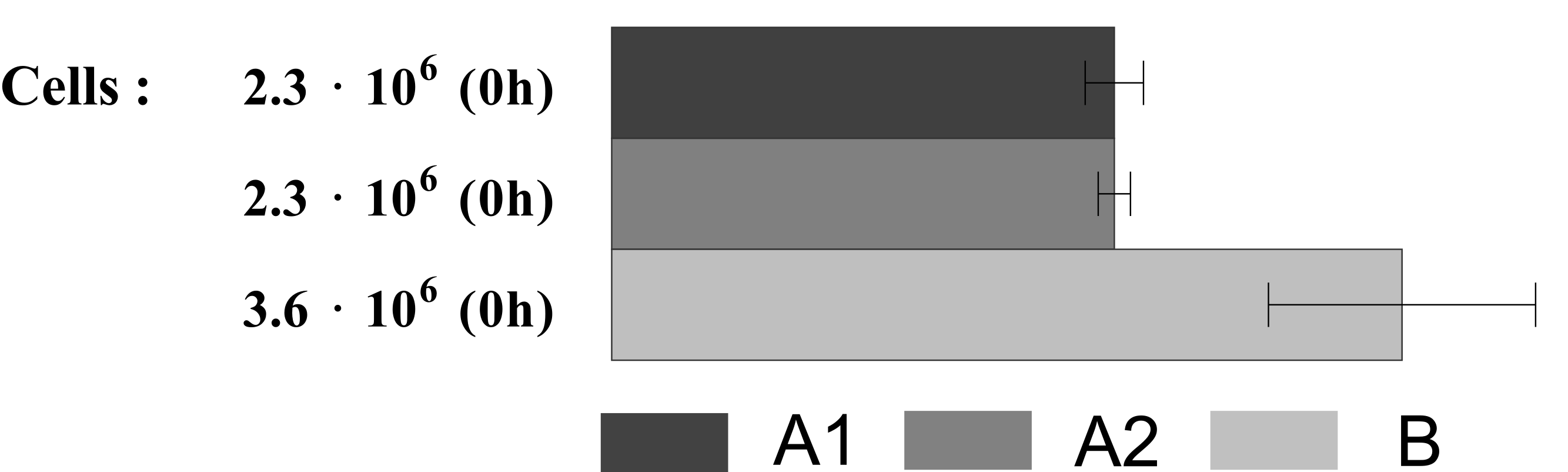

Supplement: S2 Fig — (TIFF) [file pcbi.1004899.s002.tiff]
