# Supplementary material for: HepatoDyn: A Dynamic Model of Hepatocyte Metabolism That Integrates 13C Isotopomer Data
Source: PLoS Comput Biol. 2016 Apr 28;12(4):e1004899. doi: 10.1371/journal.pcbi.1004899 (PMC4849781; doi:10.1371/journal.pcbi.1004899)
Supplement: S1 Table — This table describes all metabolites included in the model and provides abbreviation, full name, initial concentrations used as initial values for simulations and whether they are dependent variables or assumed as constants. For initial concentrations, “A1, “A2” and “B” superscripts refer to values specific for conditions A1 & A2 and B, respectively, as described in the main text. (PDF) [file pcbi.1004899.s010.pdf]

S1 Table: Metabolites included in the model. This table describes all metabolites included in the model and provides abbreviation, full name, initial concentrations used as initial values for simulations and whether they are dependent variables or assumed as constants. For initial concentrations, “A1, “A2” and “B” superscripts refer to values specific for conditions A1 & A2 and B, respectively, as described in the main text.

Species ID	Species Name	Initial Concentration	Variable
**AMP**	AMP	0.16 [1]	Yes
**bPG13**	1,3-Biphosphoglycerate	0.05	Yes
**cACoA**	Acetyl-CoA (Cytosolic)	0.04 [2]	Yes
**cADP**	ADP(Cytosolic)	0.5 [3]	Yes
**cAsp**	Aspartate (Cytosolic)	1 [2]	Yes
**cATP**	ATP(cytosolic)	3 [3]	Yes
**cCit**	Citrate (Cytosolic)	0.375 [2]	Yes
**cCoA**	Coenzyme A (Cytosolic)	0.18 [2]	Yes
**cFru**	Fructose (Cytosolic)	1	Yes
**cGDP**	GDP (Cytosolic)	0.15 [4]	Yes
**cGlc**	Glucose (Cytosolic)	10	Yes
**cGlu**	Glutamate(Cytosolic)	3.5 [2]	Yes
**cGTP**	GTP (Cytosolic)	0.5 [4]	Yes
**cKg**	α-Ketoglutarate (Cytosolic)	0.202 [2]	Yes
**cLac**	Lactate (Cytosolic)	2.3 [2]	Yes
**cMal**	Malate (Cytosolic)	0.5 [2]	Yes
**cNAD**	NAD+ (Cytosolic)	1 [5]	Yes
**cNADH**	NADH (Cytosolic)	0.002 [5]	Yes
**CO2**	CO2	5 [1]	No
**cOAA**	Oxaloacetate (Cytosolic)	0.01 [2]	Yes
**CoQ**	Ubiquinone	0.1 [6]	Yes
**CoQH**	Ubiquinol	0.6 [6]	Yes
**cPalm**	Palmitate	0.42	No
**cPi**	Phosphate (Cytosolic)	4.8 [2]	Yes
**cPyr**	Pyruvate (Cytosolic)	0.19 [2]	Yes
**DhaP**	Dihydroxyacetone	0.05 [2]	Yes
**E4P**	Eritrhose-4-phosphate	0.004 [7]	Yes
**eFru**	Fructose(Extracellular)	$3^{A1,A2}$,18.9^{B}	Yes
**eGluc**	Glucose(Extracellular)	$21.31^{A1,A2}$,18.9^{B}	Yes
**eGlut**	Glutamate(Extracellular)	0	Yes
**eLac**	Lactate (Extracellular)	0	Yes
**ePi**	Phosphate (Extracellular)	4.8	No
**ePyr**	Pyruvate (Extracellular)	0	Yes
**Fru16bP**	Fructose 1,6-bisphosphate	0.025 [2]	Yes
**Fru1P**	Fructose 1-phosphate	0.0001	Yes
**Fru26bPa**	Fructose 2,6-bisphosphate (Pool A)	0.004 [1]	Yes
**Fru26bPb**	Fructose 2,6-bisphosphate (Pool B)	0.004 [1]	Yes
**Fru6Pa**	Fructose 6-phosphate (Pool A)	0.1 [2]	Yes
**Fru6Pb**	Fructose 6-phosphate (Pool B)	0.1 [2]	Yes
**Fum**	Fumarate	0.1 [2]	Yes

**Glc1P**	Glucose 1-phosphate	0.016 [2]	Yes
**Glc6Pa**	Glucose 6-phosphate (Pool A)	0.25 [2]	Yes
**Glc6Pb**	Glucose 6-phosphate (Pool B)	0.25 [2]	Yes
**Glyc3P**	Glycerol 3-phosphate	1 [2]	Yes
**GlyGlc**	Glycogen (Extracellular Glucose equivalents)	0	Yes
**Gra**	Glyceraldehyde	0.005	Yes
**GraP**	Glyceraldehyde-3-phosphate	0.016 [2]	Yes
**mACoA**	Acetyl-CoA (Mitochondrial)	0.04 [2]	Yes
**mADP**	ADP(Mitochondrial)	1.3 [3]	Yes
**MalCoA**	Malonyl-CoA	0.004	Yes
**mAsp**	Aspartate(Mitochondrial)	1 [2]	Yes
**mATP**	ATP(Mitochondrial)	2.5 [3]	Yes
**mCit**	Citrate(Mitochondrial)	0.375 [2]	Yes
**mCoA**	Coenzyme A (Mitochondrial)	0.18 [2]	Yes
**mGDP**	GDP(Mitochondrial)	0.15[4]	Yes
**mGlu**	Glutamate(Mitochondrial)	3.5 [2]	Yes
**mGTP**	GTP(Mitochondrial)	0.5 [4]	Yes
**miCit**	Isocitrate	0.03 [2]	Yes
**mKg**	α-Ketoglutarate (Mitochondrial)	0.202 [2]	Yes
**mMal**	Malate(Mitochondrial)	0.5 [2]	Yes
**mNAD**	NAD+ (Mitochondrial)	1 [5]	Yes
**mNADH**	NADH+ (Mitochondrial)	0.2 [5]	Yes
**mOAA**	Oxaloacetate(Mitochondrial)	0.01 [2]	Yes
**mPi**	Phosphate(Mitochondrial)	5 [1]	Yes
**mPyr**	Pyruvate (Mitochondrial)	0.19 [2]	Yes
**NADP**	NADP	0.1 [5]	Yes
**NADPH**	NADPH	0.4 [5]	Yes
**PEP**	Phosphoenolpyruvate	0.14 [2]	Yes
**PG2**	2-Phosphoglycerate	0.05 [2]	Yes
**PG3**	3-Phosphoglycerate	0.4 [2]	Yes
**PGn**	6-Phosphogluconate	0.018 [7]	Yes
**PPi**	Pyrophosphate	0.014 [2]	Yes
**Rib5P**	Ribose 5-phosphate	0.009[7]	Yes
**Rul5P**	Ribulose 5-phosphate	0.012 [7]	Yes
**Sed7P**	Sedoheptulose 7-phosphate	0.068 [7]	Yes
**Suc**	Succinate	1[2]	Yes
**SuCoA**	Succinyl-CoA	0.24	Yes
**UDP**	UDP	0.15 [4]	Yes
**UDPGlc**	UDP-glucose	0.33[2]	Yes
**UTP**	UTP	0.4 [4]	Yes
**Xyl5P**	Xylulose 5-phosphate	0.018 [7]	Yes

References

1. Konig M, Bulik S, Holzhutter HG (2012) Quantifying the contribution of the liver to glucose homeostasis: a detailed kinetic model of human hepatic glucose metabolism. PLoS Comput Biol 8: e1002577.
2. Albe KR, Butler MH, Wright BE (1990) Cellular concentrations of enzymes and their substrates. J Theor Biol 143: 163-195.
3. Soboll S, Akerboom TP, Schwenke WD, Haase R, Sies H (1980) Mitochondrial and cytosolic ATP/ADP ratios in isolated hepatocytes. A comparison of the digitonin method and the non-aqueous fractionation procedure. The Biochemical journal 192: 951-954.
4. Jackson RC, Morris HP, Weber G (1977) Partial purification, properties and regulation of inosine 5'phosphate dehydrogenase in normal and malignant rat tissues. The Biochemical journal 166: 1-10.
5. Williamson DH, Lund P, Krebs HA (1967) The redox state of free nicotinamide-adenine dinucleotide in the cytoplasm and mitochondria of rat liver. The Biochemical journal 103: 514-527.
6. Miles L, Miles MV, Tang PH, Horn PS, Quinlan JG, et al. (2005) Ubiquinol: a potential biomarker for tissue energy requirements and oxidative stress. Clin Chim Acta 360: 87-96.
7. Sabate L, Franco R, Canela EI, Centelles JJ, Cascante M (1995) A model of the pentose phosphate pathway in rat liver cells. Mol Cell Biochem 142: 9-17.
