# Supplementary material for: HepatoDyn: A Dynamic Model of Hepatocyte Metabolism That Integrates 13C Isotopomer Data
Source: PLoS Comput Biol. 2016 Apr 28;12(4):e1004899. doi: 10.1371/journal.pcbi.1004899 (PMC4849781; doi:10.1371/journal.pcbi.1004899)
Supplement: S2 Table — This table describes all the reactions included in the metabolic network used in the model and indicates abbreviation, full name and stoichiometry. In reaction stoichiometry, ↔ denotes reversible reactions and → denotes irreversible reactions. (PDF) [file pcbi.1004899.s011.pdf]

S2 Table. Reactions included in the model. This table describes all the reactions included in the metabolic network used in the model and indicates abbreviation, full name and stoichiometry. In reaction stoichiometry, ↔ denotes reversible reactions and → denotes irreversible reactions.

Reaction id	Reaction name	Reaction stoichiometry
**aatc**	Aspartate aminotransferase (Cytosolic)	cAsp + cKg ↔ cGlu + cOAA
**aatm**	Aspartate aminotransferase (Mitochondrial)	mAsp + mKg ↔ mGlu + mOAA
**aco**	Aconitase	mCit ↔ miCit
**acoacar**	Acetyl-CoA carboxylase	cACoA + cATP + CO2 → cADP + cPi + MalCoA
**adk**	Adenylate kinase	AMP + cATP ↔ 2cADP
**aldo1**	Aldolase (1)	Fru16bP ↔ DhaP + GraP
**aldo2**	Aldolase (2)	Fru1P ↔ DhaP + Gra
**aldo3**	Aldolase (3)	Fru16bP + Gra ↔ Fru1P + GraP
**aldo_inv1**	Aldolase invisible (1)	Fru16bP + GraP ↔ Fru16bP + GraP
**aldo_inv2**	Aldolase invisible (2)	Fru1P + Gra ↔ Fru1P + Gra
**aspglumtrans**	Aspartate/Glutamate carrier	cGlu + mAsp ↔ cAsp + mGlu
**atpase**	Atpase	cATP → cADP + cPi
**atpmtrans**	Mitochondrial ATP/ADP carrier	cADP + mATP ↔ cATP + mADP
**box**	β-Oxidation	cATP + 7CoQ + cPalm + 8mCoA + 7mNAD → AMP + 7CoQH + 8mACoA + 7mNADH + PPi
**citly**	Citrate lyase	cATP + cCit + cCoA ↔ cACoA + cADP + cOAA + cPi
**citmtr**	Citrate carrier	cMal + mCit ↔ cCit + mMal
**cmdh**	Malate dehydrogenase (Cytosolic)	cMal + cNAD ↔ cNADH + cOAA
**cndk1**	Nucleoside diphosphate kinase 1(Cytosolic)	cATP + cGDP ↔ cADP + cGTP
**cndk2**	Nucleoside diphosphate kinase 2(Cytosolic)	cATP + UDP ↔ cADP + UTP
**coqhoxi**	Ubiquinol Oxidase	CoQH + 2mADP + 2mPi → CoQ + 2mATP
**cs**	Citrate synthase	mACoA + mOAA → mCit + mCoA
**dic**	Dicarboxylate Carrier	cPi + mMal ↔ cMal + mPi
**eno**	Enolase	PG2 ↔ PEP
**fasyn**	Fatty acid synthesis	cACoA + 7MalCoA + 14NADPH → 8cCoA + cPalm + 14NADP
**fbasea1**	Fructose 1,6-bisphosphatase (Pool A)	Fru16bP → cPi + Fru6Pa
**fbasea2**	Fructose 2,6-bisphosphatase (Pool A)	Fru26bPa → cPi + Fru6Pa
**fbaseb1**	Fructose 1,6-bisphosphatase (Pool B)	Fru16bP → cPi + Fru6Pb
**fbaseb2**	Fructose 2,6-bisphosphatase (Pool B)	Fru26bPb → cPi + Fru6Pb
**fh**	Fumarate Hydratase	Fum ↔ mMal
**fruhk**	Fructokinase	cATP + cFru → cADP + Fru1P
**frutr**	Fructose carrier	eFru ↔ cFru
**g6pasea**	Glucose-6-Phosphatase (Pool A)	Glc6Pa → cGlc + cPi

**g6paseb**	Glucose-6-Phosphatase (Pool B)	Glc6Pb → cGlc + cPi
**g6pdh**	Glucose-6-Phosphate deshydrogenase	Glc6Pa + NADP → NADPH + PGn
**gapdh**	Glyceraldehyde 3-phosphate dehydrogenase	cNAD + cPi + GraP ↔ bPG13 + cNADH
**gka**	Glucokinase (Pool A)	cATP + cGlc → cADP + Glc6Pa
**gkb**	Glucokinase (Pool B)	cATP + cGlc → cADP + Glc6Pb
**glctr**	Glucose carrier	eGluc ↔ cGlc
**glutr**	Glutamate carrier	eGlut ↔ cGlu
**glyc3pcdh**	Glycerol-3-phosphate dehydrogenase (NAD)	cNAD + Glyc3P ↔ cNADH + DhaP
**glyc3pmdh**	Glycerol-3-phosphate dehydrogenase (Ubiquinone)	CoQ + Glyc3P → CoQH + DhaP
**gp**	Glycogen Phosphorylase	cPi + GlyGlc → Glc1P
**gpia**	Glucose-6-Phosphate-Isomerase (Pool A)	Glc6Pa ↔ Fru6Pa
**gpib**	Glucose-6-Phosphate-Isomerase (Pool A)	Glc6Pb ↔ Fru6Pb
**gs**	Glycogen synthase	UDPGlc → GlyGlc + UDP
**idh**	Isocitrate dehydrogenase	miCit + mNAD ↔ CO2 + mKg + mNADH
**kdh**	α-Ketoglutarate dehydrogenase	mCoA + mKg + mNAD ↔ CO2 + mNADH + SuCoA
**lactr**	Lactate carrier	eLac ↔ cLac
**ldh**	Lactate dehydrogenase	cLac + cNAD ↔ cNADH + cPyr
**malic**	Malic enzyme	cMal + NADP ↔ CO2 + cPyr + NADPH
**malkgmtrans**	α-Ketoglutarate/Malate carrier	cMal + mKg ↔ cKg + mMal
**mmdh**	Malate dehydrogenase (Mitochondrial)	mMal + mNAD ↔ mNADH + mOAA
**mndk**	Nucleoside diphosphate kinase (Mitochondrial)	mATP + mGDP ↔ mADP + mGTP
**mpyrtr**	Mitochondrial pyruvate carrier	cPyr ↔ mPyr
**nadhdh**	NADH dehydrogenase	CoQ + mADP + mNADH + mPi → CoQH + mATP + mNAD
**pc**	Pyruvate Carboxylase	CO2 + mATP + mPyr ↔ mADP + mOAA + mPi
**pdh**	Pyruvate dehydrogenase	mCoA + mNAD + mPyr → CO2 + mACoA + mNADH
**pepck**	Phosphoenolpyruvate carboxykinase	cGTP + cOAA ↔ cGDP + CO2 + PEP
**pfkla1**	Phosphofructokinase 1(Pool A)	cATP + Fru6Pa → cADP + Fru16bP
**pfkla2**	Phosphofructokinase-2 (Pool A)	cATP + Fru6Pa → cADP + Fru26bPa
**pfklb1**	Phosphofructokinase 1(Pool B)	cATP + Fru6Pb → cADP + Fru16bP
**pfklb2**	Phosphofructokinase-2 (Pool B)	cATP + Fru6Pb → cADP + Fru26bPb
**pgk**	Phosphoglycerate kinase	bPG13 + cADP ↔ cATP + PG3
**pglm**	Phosphoglucomutase	Glc1P ↔ Glc6Pb
**pgm**	Phosphoglycerate mutase	PG3 ↔ PG2
**pgndh**	Phosphogluconate dehydrogenase	NADP + PGn → CO2 + NADPH + Rul5P

**pimtr**	Phosphate mitochondrial carrier	cPi ↔ mPi
**pitr**	Phosphate carrier	ePi ↔ cPi
**pyrtr**	Pyruvate extracellular carrier	ePyr ↔ cPyr
**pk**	Pyruvate kinase	cADP + PEP → cATP + cPyr
**ppase**	Pyrophosphatase	PPi → 2cPi
**rpi**	Ribose-5-phosphate isomerase	Rul5P ↔ Rib5P
**rul5pepi**	Ribulose-5-phosphate 4-epimerase	Rul5P ↔ Xyl5P
**scs**	Succinyl-CoA synthetase	mGDP + mPi + SuCoA ↔ mCoA + mGTP + Suc
**sdh**	Succinate dehydrogenase	CoQ + Suc ↔ CoQH + Fum
**ta**	Transaldolase	E4P + Fru6Pa ↔ GraP + Sed7P
**ta_inv1**	Transaldolase invisible (1)	Fru6Pa + GraP ↔ Fru6Pa + GraP
**ta_inv2**	Transaldolase invisible (2)	E4P + Sed7P ↔ E4P + Sed7P
**tim**	Triose-phosphate isomerase	DhaP ↔ GraP
**tk1**	Transketolase (1)	Xyl5P + Rib5P ↔ GraP + Sed7P
**tk2**	Transketolase (2)	GraP + Fru6Pa ↔ Xyl5P + E4P
**tk3**	Transketolase (3)	Fru6Pa + Rib5P ↔ E4P + Sed7P
**tk_inv1**	Transketolase invisible (1)	Xyl5P + Grap ↔ Xyl5P + Grap
**tk_inv2**	Transketolase invisible (2)	Fru6Pa + E4P ↔ Fru6Pa + E4P
**tk_inv3**	Transketolase invisible (3)	Rib5P + Sed7P ↔ Rib5P + Sed7P
**transa**	Transaminase	cKg ↔ cGlu
**trik**	Triokinase	cATP + Gra → cADP + GraP
**ugt**	UDP-glucuronosyltransferase	Glc1P + UTP ↔ PPi + UDPGlc
