# Supplementary material for: HepatoDyn: A Dynamic Model of Hepatocyte Metabolism That Integrates 13C Isotopomer Data
Source: PLoS Comput Biol. 2016 Apr 28;12(4):e1004899. doi: 10.1371/journal.pcbi.1004899 (PMC4849781; doi:10.1371/journal.pcbi.1004899)
Supplement: S3 Table — This table describes the three compartments included in the model and their volumes. (PDF) [file pcbi.1004899.s012.pdf]

S3 Table: Compartments included in the model. This table describes the three compartments included in the model and their volumes.

Compartment	Volume	Reference
**Cytosol**	$1.5 \cdot 10^{-12} \frac{l}{cell}$	[1, 2]
**Mitochondria**	$7.5 \cdot 10^{-13} \frac{l}{cell}$	[1, 2]
**Extracellular**	$1\, l$	

References

1. Uhal BD, Roehrig KL. Effect of dietary state on hepatocyte size. Biosci Rep. 1982;2(12):1003-7. Epub 1982/12/01. PubMed PMID: 7165791.
2. Kuntz E. Hepatology, textbook and atlas. 3rd ed. New York: Springer; 2008.
