# Supplementary material for: HepatoDyn: A Dynamic Model of Hepatocyte Metabolism That Integrates 13C Isotopomer Data
Source: PLoS Comput Biol. 2016 Apr 28;12(4):e1004899. doi: 10.1371/journal.pcbi.1004899 (PMC4849781; doi:10.1371/journal.pcbi.1004899)
Supplement: S4 Table — This table describes all the parameters of the kinetic model sorted by the reaction to which they are associated. (PDF) [file pcbi.1004899.s013.pdf]

S4 Table. Parameters of the kinetic model. This table describes all the parameters of the kinetic model sorted by the reaction(s) to which they are associated.

Reaction(s) id	Reaction(s) name	Parameter	Value	Units	Reference
**aatc**	Aspartate aminotransferase (Cytosolic)	V	Fitted	pmol·min^{-1}·cell^{-1}	
**aatc**	Aspartate aminotransferase (Cytosolic)	Keq	0.151515	_	[1]
**aatc**	Aspartate aminotransferase (Cytosolic)	kAsp	0.35	mM	[2]
**aatc**	Aspartate aminotransferase (Cytosolic)	kiAsp	1.6	mM	[2]
**aatc**	Aspartate aminotransferase (Cytosolic)	kKg	1.25	mM	
**aatc**	Aspartate aminotransferase (Cytosolic)	kOAA	2.05	mM	[2]
**aatc**	Aspartate aminotransferase (Cytosolic)	kiOAA	2.05	mM	
**aatc**	Aspartate aminotransferase (Cytosolic)	kGlu	0.38	mM	[2]
**aatc**	Aspartate aminotransferase (Cytosolic)	kiGlu	2.4	mM	[2]
**aatm**	Aspartate aminotransferase (Mitochondrial)	V	Fitted	pmol·min^{-1}·cell^{-1}	
**aatm**	Aspartate aminotransferase (Mitochondrial)	Keq	0.151515	_	[1]
**aatm**	Aspartate aminotransferase (Mitochondrial)	kAsp	0.75	mM	[2]
**aatm**	Aspartate aminotransferase (Mitochondrial)	kiAsp	1.9	mM	[2]
**aatm**	Aspartate aminotransferase (Mitochondrial)	kKg	1.75	mM	[2]
**aatm**	Aspartate aminotransferase (Mitochondrial)	kOAA	1.85	mM	[2]
**aatm**	Aspartate aminotransferase (Mitochondrial)	kiOAA	1.85	mM	
**aatm**	Aspartate aminotransferase (Mitochondrial)	kGlu	0.48	mM	[2]
**aatm**	Aspartate aminotransferase (Mitochondrial)	kiGlu	1.8	mM	[2]
**aco**	Aconitase	V	2	pmol·min^{-1}·cell^{-1}	
**aco**	Aconitase	KCit	0.48	mM	[3]
**aco**	Aconitase	KiCit	0.12	mM	[3]
**aco**	Aconitase	Keq	0.556	_	[1]
**acoacar**	Acetyl-CoA carboxylase	V	Fitted	pmol·min^{-1}·cell^{-1}	

**acoacar**	Acetyl-CoA carboxylase	KATP	0.015	mM	[4]
**acoacar**	Acetyl-CoA carboxylase	KACoA	0.025	mM	[4]
**acoacar**	Acetyl-CoA carboxylase	KCO2	2.5	mM	[4]
**acoacar**	Acetyl-CoA carboxylase	KCit	0.8	mM	
**acoacar**	Acetyl-CoA carboxylase	KiPalm	0.4	mM	
**adk**	Adenylate kinase	V	2	$pmol \cdot min^{-1} \cdot cell^{-1}$	
**adk**	Adenylate kinase	Keq	0.95	_	[5]
**adk**	Adenylate kinase	KiAMP	0.18	mM	[6]
**adk**	Adenylate kinase	KATP	0.23	mM	[6]
**adk**	Adenylate kinase	KiATP	0.23	mM	
**adk**	Adenylate kinase	KADP	0.15	mM	[6]
**adk**	Adenylate kinase	KiADP	0.15	mM	
**aldo1/ aldo2/aldo3/aldo_inv1/ aldo_inv2**	Aldolase (1) / Aldolase (2) / Aldolase (3) / Aldolase invisible (1) /Aldolase invisible (3)	E0	Fitted	$pmol \cdot cell^{-1}$	
**aldo1/ aldo2/aldo3/aldo_inv1 /aldo_inv2**	Aldolase (1) / Aldolase (2) / Aldolase (3) / Aldolase invisible (1) /Aldolase invisible (3)	K1	4405.64	min^{-1}	
**aldo1/ aldo2/aldo3/aldo_inv1 /aldo_inv2**	Aldolase (1) / Aldolase (2) / Aldolase (3) / Aldolase invisible (1) /Aldolase invisible (3)	K2	10	min^{-1}	
**aldo1/ aldo2/aldo3/aldo_inv1 /aldo_inv2**	Aldolase (1) / Aldolase (2) / Aldolase (3) / Aldolase invisible (1) /Aldolase invisible (3)	K3	10	min^{-1}	
**aldo1/ aldo2/aldo3/aldo_inv1/ aldo_inv2**	Aldolase (1) / Aldolase (2) / Aldolase (3) / Aldolase invisible (1) /Aldolase invisible (3)	K4	2.1896	min^{-1}	
**aldo1/ aldo2/aldo3/aldo_inv1/**	Aldolase (1) / Aldolase (2) / Aldolase (3) / Aldolase invisible (1) /Aldolase invisible (3)	K5	10	min^{-1}	

**aldo_inv2**					
**aldo1/ aldo2/aldo3/aldo_inv1/ aldo_inv2**	Aldolase (1) / Aldolase (2) / Aldolase (3) / Aldolase invisible (1) /Aldolase invisible (3)	Kr1	4.09804	min^{-1}	
**aldo1/ aldo2/aldo3/aldo_inv1/ aldo_inv2**	Aldolase (1) / Aldolase (2) / Aldolase (3) / Aldolase invisible (1) /Aldolase invisible (3)	Kr2	1985.64	min^{-1}	
**aldo1/ aldo2/aldo3/aldo_inv1 /aldo_inv2**	Aldolase (1) / Aldolase (2) / Aldolase (3) / Aldolase invisible (1) /Aldolase invisible (3)	Kr3	112.583	min^{-1}	
**aldo1/ aldo2/aldo3/aldo_inv1 /aldo_inv2**	Aldolase (1) / Aldolase (2) / Aldolase (3) / Aldolase invisible (1) /Aldolase invisible (3)	Kr4	0.0721817	min^{-1}	
**aldo1/ aldo2/aldo3/aldo_inv1 /aldo_inv2**	Aldolase (1) / Aldolase (2) / Aldolase (3) / Aldolase invisible (1) /Aldolase invisible (3)	Kr5	1985.64	min^{-1}	
**aspglumtrans**	Aspartate/Glutamate carrier	V	Fitted	$pmol\cdot min^{-1}\cdot cell^{-1}$	
**aspglumtrans**	Aspartate/Glutamate carrier	KimAsp	2.8	mM	[7]
**aspglumtrans**	Aspartate/Glutamate carrier	KcGlu	0.25	mM	[7]
**aspglumtrans**	Aspartate/Glutamate carrier	KicGlu	0.25	mM	
**aspglumtrans**	Aspartate/Glutamate carrier	KmGlu	3	mM	[7]
**aspglumtrans**	Aspartate/Glutamate carrier	KimGlu	3	mM	
**aspglumtrans**	Aspartate/Glutamate carrier	KicAsp	0.12	mM	[7]
**aspglumtrans**	Aspartate/Glutamate carrier	Keq	1	_	
**atpase**	Atpase	V	Fitted	$pmol\cdot min^{-1}\cdot cell^{-1}$	
**atpase**	Atpase	KATP	0.5	mM	
**atpmtrans**	Mitochondrial ATP/ADP carrier	V	Fitted	$pmol\cdot min^{-1}\cdot cell^{-1}$	

**atpmtrans**	Mitochondrial ATP/ADP carrier	Keq	10	_	
**atpmtrans**	Mitochondrial ATP/ADP carrier	KmATP	0.75	mM	
**atpmtrans**	Mitochondrial ATP/ADP carrier	KimATP	0.75	mM	
**atpmtrans**	Mitochondrial ATP/ADP carrier	KcADP	0.75	mM	
**atpmtrans**	Mitochondrial ATP/ADP carrier	kcATP	0.75	mM	
**atpmtrans**	Mitochondrial ATP/ADP carrier	kicATP	0.75	mM	
**atpmtrans**	Mitochondrial ATP/ADP carrier	kmADP	0.75	mM	
**atpmtrans**	Mitochondrial ATP/ADP carrier	kimADP	0.75	mM	
**box**	β-Oxidation	V	Fitted	$pmol \cdot min^{-1} \cdot cell^{-1}$	
**box**	β-Oxidation	KPalm	0.05	mM	
**box**	β-Oxidation	KATP	0.1	mM	
**box**	β-Oxidation	KCoA	0.001	mM	
**box**	β-Oxidation	KCoQ	0.0005	mM	
**box**	β-Oxidation	KNAD	0.075	mM	
**box**	β-Oxidation	KiMalCoA	0.05	mM	
**citly**	Citrate lyase	V	Fitted	$pmol \cdot min^{-1} \cdot cell^{-1}$	
**citly**	Citrate lyase	Keq	1000	mM	[1]
**citly**	Citrate lyase	KCit	0.11	mM	[8]
**citly**	Citrate lyase	KATP	0.16	mM	[8]
**citly**	Citrate lyase	KCoA	0.004	mM	[8]
**citly**	Citrate lyase	KACoA	0.001	mM	
**citly**	Citrate lyase	KOAA	0.01	mM	
**citly**	Citrate lyase	KADP	0.16	mM	
**citly**	Citrate lyase	KPi	1	mM	
**citmtr**	Citrate carrier	V	Fitted	$pmol \cdot min^{-1} \cdot cell^{-1}$	
**citmtr**	Citrate carrier	Keq	0.1	mM	

**citmtr**	Citrate carrier	KimCit	0.027	mM	[7]
**citmtr**	Citrate carrier	KcMal	0.25	mM	[7]
**citmtr**	Citrate carrier	KicMal	0.25	mM	
**citmtr**	Citrate carrier	KmMal	0.06	mM	[7]
**citmtr**	Citrate carrier	KimMal	0.06	mM	
**citmtr**	Citrate carrier	KicCit	0.032	mM	[7]
**cmdh**	Malate dehydrogenase (Cytosolic)	V	Fitted	$pmol \cdot min^{-1} \cdot cell^{-1}$	
**cmdh**	Malate dehydrogenase (Cytosolic)	Keq	0.000028	_	[1]
**cmdh**	Malate dehydrogenase (Cytosolic)	kNAD	0.14	mM	[9]
**cmdh**	Malate dehydrogenase (Cytosolic)	kiNAD	0.14	mM	
**cmdh**	Malate dehydrogenase (Cytosolic)	kMal	8	mM	[10]
**cmdh**	Malate dehydrogenase (Cytosolic)	kiMal	8	mM	
**cmdh**	Malate dehydrogenase (Cytosolic)	kOAA	0.0083	mM	[11]
**cmdh**	Malate dehydrogenase (Cytosolic)	kiOAA	0.0083	mM	
**cmdh**	Malate dehydrogenase (Cytosolic)	kNADH	0.0205	mM	[11]
**cmdh**	Malate dehydrogenase (Cytosolic)	kiNADH	0.0205	mM	
**cndk1/ cndk2**	Nucleoside diphosphate kinase 1(Cytosolic) / Nucleoside diphosphate kinase 2(Cytosolic)	V	2	$pmol \cdot min^{-1} \cdot cell^{-1}$	
**cndk1/ cndk2**	Nucleoside diphosphate kinase 1(Cytosolic) / Nucleoside diphosphate kinase 2(Cytosolic)	Keq	1	_	[1]
**cndk1/ cndk2**	Nucleoside diphosphate kinase 1(Cytosolic) / Nucleoside diphosphate kinase 2(Cytosolic)	KATP	1.33	mM	[5]
**cndk1/ cndk2**	Nucleoside diphosphate kinase 1(Cytosolic) / Nucleoside diphosphate kinase 2(Cytosolic)	KiATP	1.33	mM	
**cndk1/ cndk2**	Nucleoside diphosphate kinase 1(Cytosolic) / Nucleoside diphosphate kinase 2(Cytosolic)	KGDP	0.031	mM	[5]
**cndk1/ cndk2**	Nucleoside diphosphate kinase 1(Cytosolic) / Nucleoside diphosphate kinase 2(Cytosolic)	KADP	0.042	mM	[5]

**cndk1/ cndk2**	Nucleoside diphosphate kinase 1(Cytosolic) / Nucleoside diphosphate kinase 2(Cytosolic)	KiADP	0.042	mM	
**cndk1**	Nucleoside diphosphate kinase 1(Cytosolic)	KGTP	0.15	mM	[5]
**cndk1**	Nucleoside diphosphate kinase 1(Cytosolic)	KiGTP	0.15	mM	
**cndk1/ cndk2**	Nucleoside diphosphate kinase 1(Cytosolic) / Nucleoside diphosphate kinase 2(Cytosolic)	KUDP	0.19	mM	[5]
**cndk2**	Nucleoside diphosphate kinase 2(Cytosolic)	KiUDP	0.19	mM	[5]
**cndk2**	Nucleoside diphosphate kinase 2(Cytosolic)	KUTP	16	mM	[5]
**cndk2**	Nucleoside diphosphate kinase 2(Cytosolic)	KiUTP	16	mM	
**coqhoxi**	Ubiquinol Oxidase	k	Fitted	$l^5 \cdot mmol^{-4} \cdot cell^{-1}$	
**cs**	Citrate synthase	V	Fitted	$pmol \cdot min^{-1} \cdot cell^{-1}$	
**cs**	Citrate synthase	KiACoA	0.005	mM	[12]
**cs**	Citrate synthase	KOAA	0.0059	mM	[12]
**cs**	Citrate synthase	KiOAA	0.0059	mM	
**cs**	Citrate synthase	KiCit	2	mM	
**dic**	Dicarboxylate Carrier	V	Fitted	$pmol \cdot min^{-1} \cdot cell^{-1}$	
**dic**	Dicarboxylate Carrier	Keq	1	_	
**dic**	Dicarboxylate Carrier	KimMal	0.92	mM	[7]
**dic**	Dicarboxylate Carrier	KcPi	1.41	mM	[7]
**dic**	Dicarboxylate Carrier	KicPi	1.41	mM	
**dic**	Dicarboxylate Carrier	KmPi	0.93	mM	[7]
**dic**	Dicarboxylate Carrier	KimPi	0.93	mM	
**dic**	Dicarboxylate Carrier	KicMal	0.49	mM	[7]
**eno**	Enolase	V	2	$pmol \cdot min^{-1} \cdot cell^{-1}$	
**eno**	Enolase	Keq	4	_	[1]

**eno**	Enolase	KPG2	0.14	mM	[13]
**eno**	Enolase	KPEP	0.31	mM	[13]
**fasyn**	Fatty acid synthesis	V	Fitted	$pmol \cdot min^{-1} \cdot cell^{-1}$	
**fasyn**	Fatty acid synthesis	KMalCoA	0.0019	mM	[14]
**fasyn**	Fatty acid synthesis	KACoA	0.0039	mM	[14]
**fasyn**	Fatty acid synthesis	KNADPH	0.01	mM	
**fbasea1**	Fructose 1,6-bisphosphatase (Pool A)	V	Fitted	$pmol \cdot min^{-1} \cdot cell^{-1}$	
**fbasea1/ fbaseb1**	Fructose 1,6-bisphosphatase (Pool A) / Fructose 1,6-bisphosphatase (Pool B)	KFru16bp	0.0055	mM	[15]
**fbasea1/ fbaseb1**	Fructose 1,6-bisphosphatase (Pool A) / Fructose 1,6-bisphosphatase (Pool B)	kAMP	0.24	mM	[15]
**fbasea1/ fbaseb1**	Fructose 1,6-bisphosphatase (Pool A) / Fructose 1,6-bisphosphatase (Pool B)	nAMP	1	_	
**fbasea1/ fbaseb1**	Fructose 1,6-bisphosphatase (Pool A) / Fructose 1,6-bisphosphatase (Pool B)	KiFru26bp	0.001	mM	[5]
**fbasea2/ pfkla2**	Fructose 1,6-bisphosphatase (Pool A) / Phosphofructokinase-2 (Pool A)	V	0.0001	$pmol \cdot min^{-1} \cdot cell^{-1}$	
**fbasea2/ fbaseb2**	Fructose 2,6-bisphosphatase (Pool A) / Fructose 2,6-bisphosphatase (Pool B)	Pfk2Fb2Factor	0.4	_	[16]
**fbasea2/ fbaseb2**	Fructose 2,6-bisphosphatase (Pool A) / Fructose 2,6-bisphosphatase (Pool B)	p	0.7	_	[5]
**fbasea2/ fbaseb2**	Fructose 2,6-bisphosphatase (Pool A) / Fructose 2,6-bisphosphatase (Pool B)	KdpFru26bP	0.01	mM	[5]
**fbasea2/ fbaseb2**	Fructose 2,6-bisphosphatase (Pool A) / Fructose 2,6-bisphosphatase (Pool B)	KpFru26bP	0.005	mM	[5]
**fbasea2/ fbaseb2**	Fructose 2,6-bisphosphatase (Pool A) / Fructose 2,6-bisphosphatase (Pool B)	KidpFru6P	0.0035	mM	[5]

**fbasea2/ fbaseb2**	Fructose 2,6-bisphosphatase (Pool A) / Fructose 2,6-bisphosphatase (Pool B)	KipFru6p	0.05	mM	[5]
**fbaseb1**	Fructose 1,6-bisphosphatase (Pool B)	V	Fitted	$pmol·min^{-1}·cell^{-1}$	
**fbaseb2/ pfklb2**	Fructose 1,6-bisphosphatase (Pool B) / Phosphofructokinase-2 (Pool B)	V	0.0001	$pmol·min^{-1}·cell^{-1}$	
**fh**	Fumarate Hydratase	V	Fitted	$pmol·min^{-1}·cell^{-1}$	
**fh**	Fumarate Hydratase	Keq	12	_	[1]
**fh**	Fumarate Hydratase	KFum	0.005	mM	[17]
**fh**	Fumarate Hydratase	KMal	0.025	mM	[17]
**fruhk**	Fructokinase	V	Fitted	$pmol·min^{-1}·cell^{-1}$	
**fruhk**	Fructokinase	KiATP	0.15	mM	[18]
**fruhk**	Fructokinase	KFru	0.8	mM	[18]
**fruhk**	Fructokinase	KiFru	0.8	mM	
**frutransport**	Fructose carrier	V	Fitted	$pmol·min^{-1}·cell^{-1}$	
**frutransport**	Fructose carrier	Keq	1	_	
**frutr**	Fructose carrier	KFru	67	mM	[19]
**g6pasea**	Glucose-6-Phosphatase (Pool A)	V	Fitted	$pmol·min^{-1}·cell^{-1}$	
**g6pasea/ g6paseb**	Glucose-6-Phosphatase (Pool A) / Fructose 1,6-bisphosphatase (Pool B	KGlc6P	2	mM	[5]
**g6paseb**	Glucose-6-Phosphatase (Pool B)	V	Fitted	$pmol·min^{-1}·cell^{-1}$	
**g6pdh**	Glucose-6-Phosphate deshydrogenase	V	Fitted	$pmol·min^{-1}·cell^{-1}$	
**g6pdh**	Glucose-6-Phosphate deshydrogenase	KNADP	0.0048	mM	[20]
**g6pdh**	Glucose-6-Phosphate deshydrogenase	KiNADP	0.009	mM	[20]

**g6pdh**	Glucose-6-Phosphate deshydrogenase	KGlc6P	0.036	mM	[20]
**g6pdh**	Glucose-6-Phosphate deshydrogenase	KiGlc6P	0.036	mM	
**g6pdh**	Glucose-6-Phosphate deshydrogenase	KNADPH	0.0011	mM	
**g6pdh**	Glucose-6-Phosphate deshydrogenase	KiNADPH	0.0011	mM	[20]
**g6pdh**	Glucose-6-Phosphate deshydrogenase	KPGn	0.01	mM	
**g6pdh**	Glucose-6-Phosphate deshydrogenase	KiPGn	0.01	mM	
**gapdh**	Glyceraldehyde 3-phosphate dehydrogenase	V	2	pmol·min^{-1}·cell^{-1}	
**gapdh**	Glyceraldehyde 3-phosphate dehydrogenase	Keq	0.00015	_	[1]
**gapdh**	Glyceraldehyde 3-phosphate dehydrogenase	Kgrap	0.07	mM	[21]
**gapdh**	Glyceraldehyde 3-phosphate dehydrogenase	Knad	0.05	mM	[21]
**gapdh**	Glyceraldehyde 3-phosphate dehydrogenase	Kpi	3.9	mM	[5]
**gapdh**	Glyceraldehyde 3-phosphate dehydrogenase	Knadh	0.01	mM	[21]
**gapdh**	Glyceraldehyde 3-phosphate dehydrogenase	Kbpg13	0.14	mM	[21]
**gka**	Glucokinase (Pool A)	V	Fitted	pmol·min^{-1}·cell^{-1}	
**gka/ gkb**	Glucokinase (Pool A) / Glucokinase (Pool B)	nGlc	1.58	_	[22]
**gka/ gkb**	Glucokinase (Pool A) / Glucokinase (Pool B)	Kglc	9	mM	[22]
**gka/ gkb**	Glucokinase (Pool A) / Glucokinase (Pool B)	Katp	0.26	mM	[23]
**gka/ gkb**	Glucokinase (Pool A) / Glucokinase (Pool B)	ngkrp	2	_	[5]
**gka/ gkb**	Glucokinase (Pool A) / Glucokinase (Pool B)	bgkrp	0.7	_	[5]
**gka/ gkb**	Glucokinase (Pool A) / Glucokinase (Pool B)	KgkrpFru6p	0.01	mM	[5]
**gka/ gkb**	Glucokinase (Pool A) / Glucokinase (Pool B)	KFru1P	0.001	mM	[24]
**gka/ gkb**	Glucokinase (Pool A) / Glucokinase (Pool B)	KgkrpGlc	15	mM	[5]
**gkb**	Glucokinase (Pool B)	V	Fitted	pmol·min^{-1}·cell^{-1}	
**glctr**	Glucose carrier	V	2	pmol·min^{-1}·cell^{-1}	
**glctr**	Glucose carrier	kGlc	17	mM	[25]

**glctr**	Glucose carrier	Keq	1	mM	
**glutr**	Glutamate carrier	V	Fitted	pmol·min^{-1}·cell^{-1}	
**glutr**	Glutamate carrier	KGlu	2	mM	
**glutr**	Glutamate carrier	Keq	10	_	
**glyc3pcdh**	Glycerol-3-phosphate dehydrogenase (NAD)	V	Fitted	pmol·min^{-1}·cell^{-1}	
**glyc3pcdh**	Glycerol-3-phosphate dehydrogenase (NAD)	Keq	0.000056	_	[26]
**glyc3pcdh**	Glycerol-3-phosphate dehydrogenase (NAD)	kNAD	0.13	mM	[27]
**glyc3pcdh**	Glycerol-3-phosphate dehydrogenase (NAD)	kiNAD	0.13	mM	
**glyc3pcdh**	Glycerol-3-phosphate dehydrogenase (NAD)	KGlyc3P	0.22	mM	[27]
**glyc3pcdh**	Glycerol-3-phosphate dehydrogenase (NAD)	KiGlyc3P	0.22	mM	
**glyc3pcdh**	Glycerol-3-phosphate dehydrogenase (NAD)	KDhaP	0.16	mM	[27]
**glyc3pcdh**	Glycerol-3-phosphate dehydrogenase (NAD)	KiDhaP	0.16	mM	
**glyc3pcdh**	Glycerol-3-phosphate dehydrogenase (NAD)	kNADH	0.08	mM	[27]
**glyc3pcdh**	Glycerol-3-phosphate dehydrogenase (NAD)	kiNADH	0.08	mM	
**glyc3pmdh**	Glycerol-3-phosphate dehydrogenase (Ubiquinone)	V	Fitted	pmol·min^{-1}·cell^{-1}	
**glyc3pmdh**	Glycerol-3-phosphate dehydrogenase (Ubiquinone)	KGlyc3P	6.2	mM	[28]
**glyc3pmdh**	Glycerol-3-phosphate dehydrogenase (Ubiquinone)	KiGlyc3P	6.2	mM	
**glyc3pmdh**	Glycerol-3-phosphate dehydrogenase (Ubiquinone)	KCoQ	0.05	mM	[28]
**gp**	Glycerol-3-phosphate dehydrogenase (Ubiquinone)	V	Fitted	pmol·min^{-1}·cell^{-1}	
**gp**	Glycerol-3-phosphate dehydrogenase (Ubiquinone)	p	0.7	_	[5]
**gp**	Glycerol-3-phosphate dehydrogenase (Ubiquinone)	KDPGlyc	0.048	mM	[5]
**gp**	Glycerol-3-phosphate dehydrogenase (Ubiquinone)	KPGlyc	0.027	mM	[5]
**gp**	Glycerol-3-phosphate dehydrogenase (Ubiquinone)	KDPPi	300	mM	[5]
**gp**	Glycerol-3-phosphate dehydrogenase (Ubiquinone)	KPPi	5	mM	[5]
**gp**	Glycerol-3-phosphate dehydrogenase (Ubiquinone)	KGlcPi	5	mM	[5]

**gp**	Glycerol-3-phosphate dehydrogenase (Ubiquinone)	KDPAMPbase	0.03	mM	[5]
**gp**	Glycerol-3-phosphate dehydrogenase (Ubiquinone)	KDPAMPmax	0.3	mM	[5]
**gp**	Glycerol-3-phosphate dehydrogenase (Ubiquinone)	KDPAMPa	1	mM	[5]
**gpia**	Glucose-6-Phosphate-Isomerase (Pool A)	V	2	$pmol \cdot min^{-1} \cdot cell^{-1}$	
**gpia/ gpib**	Glucose-6-Phosphate-Isomerase (Pool A) / Glucose-6-Phosphate-Isomerase (Pool B)	Keq	0.4	_	[1]
**gpia/ gpib**	Glucose-6-Phosphate-Isomerase (Pool A) / Glucose-6-Phosphate-Isomerase (Pool B)	kGlc6p	0.445	mM	[29]
**gpia/ gpib**	Glucose-6-Phosphate-Isomerase (Pool A) / Glucose-6-Phosphate-Isomerase (Pool B)	kFru6P	0.0635	mM	[29]
**gpib**	Glucose-6-Phosphate-Isomerase (Pool B)	V	2	$pmol \cdot min^{-1} \cdot cell^{-1}$	
**gs**	Glycogen synthase	V	Fitted	$pmol \cdot min^{-1} \cdot cell^{-1}$	
**gs**	Glycogen synthase	p	0.7	_	[5]
**gs**	Glycogen synthase	K1dp	0.224	mM	[5]
**gs**	Glycogen synthase	K1p	3.003	mM	[5]
**gs**	Glycogen synthase	K2dp	0.1504	mM	[5]
**gs**	Glycogen synthase	K2p	0.09029	mM	[5]
**idh**	Isocitrate dehydrogenase	V	Fitted	$pmol \cdot min^{-1} \cdot cell^{-1}$	
**idh**	Isocitrate dehydrogenase	Keq	1200	mM	[1]
**idh**	Isocitrate dehydrogenase	KiCit	0.69	mM	[30]
**idh**	Isocitrate dehydrogenase	KNAD	0.08	mM	[30]
**idh**	Isocitrate dehydrogenase	KKg	0.69	mM	
**idh**	Isocitrate dehydrogenase	KNADH	0.08	mM	
**idh**	Isocitrate dehydrogenase	KCO2	2	mM	
**kdh**	α-Ketoglutarate dehydrogenase	V	Fitted	$pmol \cdot min^{-1} \cdot cell^{-1}$	

**kdh**	α-Ketoglutarate dehydrogenase	Keq	60000	_	[1]
**kdh**	α-Ketoglutarate dehydrogenase	KKg	0.3	mM	[31]
**kdh**	α-Ketoglutarate dehydrogenase	KNAD	0.22	mM	[32]
**kdh**	α-Ketoglutarate dehydrogenase	KCoA	0.01	mM	
**kdh**	α-Ketoglutarate dehydrogenase	KSuCoA	0.03	mM	[33]
**kdh**	α-Ketoglutarate dehydrogenase	KNADH	0.07	mM	
**kdh**	α-Ketoglutarate dehydrogenase	KCO2	2	mM	
**lactr/ pyrtr**	Lactate carrier / Pyruvate extracellular carrier	V	Fitted	$pmol \cdot min^{-1} \cdot cell^{-1}$	
**lactr/ pyrtr**	Lactate carrier / Pyruvate extracellular carrier	Keq	1	_	
**lactr/ pyrtr**	Lactate carrier / Pyruvate extracellular carrier	KLac	2.4	mM	[34]
**lactr/ pyrtr**	Lactate carrier / Pyruvate extracellular carrier	KPyr	0.6	mM	[34]
**ldh**	Lactate dehydrogenase	V	Fitted	$pmol \cdot min^{-1} \cdot cell^{-1}$	
**ldh**	Lactate dehydrogenase	Keq	0.00011	_	
**ldh**	Lactate dehydrogenase	KNAD	1.1	mM	[35]
**ldh**	Lactate dehydrogenase	KiNAD	1.1	mM	
**ldh**	Lactate dehydrogenase	KLac	11.9	mM	[36]
**ldh**	Lactate dehydrogenase	KiLac	11.9	mM	
**ldh**	Lactate dehydrogenase	KPyr	0.398	mM	[37]
**ldh**	Lactate dehydrogenase	KiPyr	0.398	mM	
**ldh**	Lactate dehydrogenase	KNADH	0.061	mM	[35]
**ldh**	Lactate dehydrogenase	KiNADH	0.061	mM	
**malic**	Malic enzyme	V	Fitted	$pmol \cdot min^{-1} \cdot cell^{-1}$	
**malic**	Malic enzyme	Keq	6	_	[1]
**malic**	Malic enzyme	KMal	0.12	mM	[38]
**malic**	Malic enzyme	kNADP	0.0092	mM	[38]

**malic**	Malic enzyme	KPyr	5.9	mM	[38]
**malic**	Malic enzyme	kNADPH	0.0053	mM	[38]
**malic**	Malic enzyme	KCO2	27	mM	[38]
**malkgmtrans**	α-Ketoglutarate/Malate carrier	V	Fitted	$pmol \cdot min^{-1} \cdot cell^{-1}$	
**malkgmtrans**	α-Ketoglutarate/Malate carrier	Keq	1	_	
**malkgmtrans**	α-Ketoglutarate/Malate carrier	KimKg	0.17	mM	[7]
**malkgmtrans**	α-Ketoglutarate/Malate carrier	KcMal	1.36	mM	[7]
**malkgmtrans**	α-Ketoglutarate/Malate carrier	KicMal	1.36	mM	[7]
**malkgmtrans**	α-Ketoglutarate/Malate carrier	KmMal	0.71	mM	[7]
**malkgmtrans**	α-Ketoglutarate/Malate carrier	KimMal	0.71	mM	[7]
**malkgmtrans**	α-Ketoglutarate/Malate carrier	KicKg	0.31	mM	[7]
**mmdh**	Malate dehydrogenase (Mitochondrial)	V	Fitted	$pmol \cdot min^{-1} \cdot cell^{-1}$	
**mmdh**	Malate dehydrogenase (Mitochondrial)	Keq	0.000028	_	[1]
**mmdh**	Malate dehydrogenase (Mitochondrial)	kNAD	0.35	mM	[39]
**mmdh**	Malate dehydrogenase (Mitochondrial)	kiNAD	0.35	mM	
**mmdh**	Malate dehydrogenase (Mitochondrial)	kMal	3.53	mM	[39]
**mmdh**	Malate dehydrogenase (Mitochondrial)	kiMal	3.53	mM	
**mmdh**	Malate dehydrogenase (Mitochondrial)	kOAA	0.0348	mM	[11]
**mmdh**	Malate dehydrogenase (Mitochondrial)	kiOAA	0.0348	mM	
**mmdh**	Malate dehydrogenase (Mitochondrial)	kNADH	0.0145	mM	[11]
**mmdh**	Malate dehydrogenase (Mitochondrial)	kiNADH	0.0145	mM	
**mndk**	Nucleoside diphosphate kinase (Mitochondrial)	V	2	$pmol \cdot min^{-1} \cdot cell^{-1}$	
**mndk**	Nucleoside diphosphate kinase (Mitochondrial)	Keq	1	_	[1]
**mndk**	Nucleoside diphosphate kinase (Mitochondrial)	KATP	1.33	mM	[5]
**mndk**	Nucleoside diphosphate kinase (Mitochondrial)	KiATP	1.33	mM	
**mndk**	Nucleoside diphosphate kinase (Mitochondrial)	KGDP	0.031	mM	[5]

**mndk**	Nucleoside diphosphate kinase (Mitochondrial)	KADP	0.042	mM	[5]
**mndk**	Nucleoside diphosphate kinase (Mitochondrial)	KiADP	0.042	mM	
**mndk**	Nucleoside diphosphate kinase (Mitochondrial)	KGTP	0.15	mM	[5]
**mndk**	Nucleoside diphosphate kinase (Mitochondrial)	KiGTP	0.15	mM	
**mpyrtr**	Mitochondrial pyruvate carrier	V	2	$pmol \cdot min^{-1} \cdot cell^{-1}$	
**mpyrtr**	Mitochondrial pyruvate carrier	Keq	1	_	
**mpyrtr**	Mitochondrial pyruvate carrier	KPyr	0.24	mM	[7]
**mpyrtr**	Mitochondrial pyruvate carrier	KiMal	10	mM	
**nadhdh**	NADH dehydrogenase	k	Fitted		
**pc**	Pyruvate Carboxylase	V	Fitted	$pmol \cdot min^{-1} \cdot cell^{-1}$	
**pc**	Pyruvate Carboxylase	Keq	180	_	[1]
**pc**	Pyruvate Carboxylase	KPyr	0.22	mM	[40]
**pc**	Pyruvate Carboxylase	KATP	0.22	mM	[40]
**pc**	Pyruvate Carboxylase	KCO2	3.2	mM	[40]
**pc**	Pyruvate Carboxylase	KOAA	0.002	mM	
**pc**	Pyruvate Carboxylase	kADP	0.22	mM	
**pc**	Pyruvate Carboxylase	KPi	3.2	mM	
**pc**	Pyruvate Carboxylase	KACoA	0.022	mM	[41]
**pc**	Pyruvate Carboxylase	beta	50	_	[42]
**pdh**	Pyruvate dehydrogenase	V	Fitted	$pmol \cdot min^{-1} \cdot cell^{-1}$	
**pdh**	Pyruvate dehydrogenase	p	0.7	_	[5]
**pdh**	Pyruvate dehydrogenase	KPyr	0.025	mM	[5]
**pdh**	Pyruvate dehydrogenase	KCoA	0.013	mM	[5]
**pdh**	Pyruvate dehydrogenase	KNAD	0.05	mM	[5]
**pdh**	Pyruvate dehydrogenase	KiACoA	0.035	mM	[5]
**pdh**	Pyruvate dehydrogenase	KiNADH	0.036	mM	[5]

**pdh**	Pyruvate dehydrogenase	alfaDP	5	_	[5]
**pdh**	Pyruvate dehydrogenase	alfaP	1	_	[5]
**pepck**	Phosphoenolpyruvate carboxykinase	V	Fitted	$pmol\cdot min^{-1}\cdot cell^{-1}$	
**pepck**	Phosphoenolpyruvate carboxykinase	Keq	300	mM	[1]
**pepck**	Phosphoenolpyruvate carboxykinase	KGTP	0.068	mM	[43]
**pepck**	Phosphoenolpyruvate carboxykinase	KOAA	0.052	mM	[43]
**pepck**	Phosphoenolpyruvate carboxykinase	KPEP	0.294	mM	[43]
**pepck**	Phosphoenolpyruvate carboxykinase	KGDP	0.039	mM	[43]
**pepck**	Phosphoenolpyruvate carboxykinase	KCO2	1.194	mM	[43]
**pfkla1**	Phosphofructokinase 1(Pool A)	V	Fitted	$pmol\cdot min^{-1}\cdot cell^{-1}$	
**pfkla1/ pfklb1**	Phosphofructokinase 1(Pool A) / Phosphofructokinase 1(Pool B)	KATP	0.111	mM	[5]
**pfkla1/ pfklb1**	Phosphofructokinase 1(Pool A) / Phosphofructokinase 1(Pool B)	KFru6P	0.077	mM	[5]
**pfkla1/ pfklb1**	Phosphofructokinase 1(Pool A) / Phosphofructokinase 1(Pool B)	KiFru6P	0.077	mM	[5]
**pfkla1/ pfklb1**	Phosphofructokinase 1(Pool A) / Phosphofructokinase 1(Pool B)	KFru26bP	0.001	mM	[5]
**pfkla2/ pfklb2**	Phosphofructokinase 2(Pool A) / Phosphofructokinase 2(Pool B)	p	0.7	_	[5]
**pfkla2/ pfklb2**	Phosphofructokinase 2(Pool A) / Phosphofructokinase 2(Pool B)	ndp	1.3	_	[5]
**pfkla2/ pfklb2**	Phosphofructokinase 2(Pool A) / Phosphofructokinase 2(Pool B)	np	2.1	_	[5]
**pfkla2/ pfklb2**	Phosphofructokinase 2(Pool A) / Phosphofructokinase 2(Pool B)	KdpATP	0.28	mM	[5]
**pfkla2/ pfklb2**	Phosphofructokinase 2(Pool A) / Phosphofructokinase 2(Pool B)	KpATP	0.65	mM	[5]

**pfkla2/ pfklb2**	Phosphofructokinase 2(Pool A) / Phosphofructokinase 2(Pool B)	KdpFru6P	0.016	mM	[5]
**pfkla2/ pfklb2**	Phosphofructokinase 2(Pool A) / Phosphofructokinase 2(Pool B)	KpFru6P	0.05	mM	[5]
**pfkla2/ pfklb2**	Phosphofructokinase 2(Pool A) / Phosphofructokinase 2(Pool B)	KiCit	0.2	mM	[44]
**pfkla2/ pfklb2**	Phosphofructokinase 2(Pool A) / Phosphofructokinase 2(Pool B)	KiPEP	0.2	mM	[44]
**pfklb1**	Phosphofructokinase 1(Pool B)	V	Fitted	$pmol \cdot min^{-1} \cdot cell^{-1}$	
**pgk**	Phosphoglycerate kinase	V	6	$pmol \cdot min^{-1} \cdot cell^{-1}$	
**pgk**	Phosphoglycerate kinase	Keq	3200	_	[1]
**pgk**	Phosphoglycerate kinase	KibPG13	0.0005	mM	[45]
**pgk**	Phosphoglycerate kinase	KADP	0.11	mM	[45]
**pgk**	Phosphoglycerate kinase	KiADP	0.11	mM	
**pgk**	Phosphoglycerate kinase	KATP	0.12	mM	[45]
**pgk**	Phosphoglycerate kinase	KiATP	0.12	mM	
**pgk**	Phosphoglycerate kinase	KiPG3	0.05	mM	[45]
**pglm**	Phosphoglucomutase	V	0.15	$pmol \cdot min^{-1} \cdot cell^{-1}$	
**pglm**	Phosphoglucomutase	Keq	19	_	[1]
**pglm**	Phosphoglucomutase	KGlc1P	0.045	mM	[5]
**pglm**	Phosphoglucomutase	KGlc6Pb	0.67	mM	[5]
**pgm**	Phosphoglycerate mutase	V	2	$pmol \cdot min^{-1} \cdot cell^{-1}$	
**pgm**	Phosphoglycerate mutase	Keq	0.125	_	[1]
**pgm**	Phosphoglycerate mutase	KPG3	0.1	mM	[46]
**pgm**	Phosphoglycerate mutase	KPG2	0.2	mM	[46]

**pgndh**	Phosphogluconate dehydrogenase	V	Fitted	$pmol\cdot min^{-1}\cdot cell^{-1}$	
**pgndh**	Phosphogluconate dehydrogenase	KNADP	0.0135	mM	[20]
**pgndh**	Phosphogluconate dehydrogenase	KiNADP	0.0048	mM	[20]
**pgndh**	Phosphogluconate dehydrogenase	KPgn	0.0292	mM	[20]
**pgndh**	Phosphogluconate dehydrogenase	KiPgn	2.176	mM	[20]
**pgndh**	Phosphogluconate dehydrogenase	KNADPH	0.00022	mM	[20]
**pgndh**	Phosphogluconate dehydrogenase	KiNADPH	0.0051	mM	[20]
**pgndh**	Phosphogluconate dehydrogenase	KRul5P	0.02	mM	[20]
**pgndh**	Phosphogluconate dehydrogenase	KiRul5P	0.02	mM	
**pimtr**	Phosphate mitochondrial carrier	V	Fitted	$pmol\cdot min^{-1}\cdot cell^{-1}$	
**pimtr**	Phosphate mitochondrial carrier	Keq	1	_	
**pimtr**	Phosphate mitochondrial carrier	KcPi	1.8	mM	[7]
**pimtr**	Phosphate mitochondrial carrier	KmPi	9.4	mM	[7]
**pitr**	Phosphate carrier	V	Fitted	$pmol\cdot min^{-1}\cdot cell^{-1}$	
**pitr**	Phosphate carrier	KPi	1	mM	
**pitr**	Phosphate carrier	Keq	1	_	
**pk**	Pyruvate kinase	V	Fitted	$pmol\cdot min^{-1}\cdot cell^{-1}$	
**pk**	Pyruvate kinase	p	0.7	_	[5]
**pk**	Pyruvate kinase	KADP	0.3	mM	[47]
**pk**	Pyruvate kinase	nDPPEP	3.5	_	[5]
**pk**	Pyruvate kinase	nPPEP	3.5	_	[5]
**pk**	Pyruvate kinase	alfaDPbase	0.08	_	[5]
**pk**	Pyruvate kinase	alfaPbase	0.04	_	[5]
**pk**	Pyruvate kinase	KDPFru16bP	0.00016	mM	[5]
**pk**	Pyruvate kinase	KPFru16bpP	0.00035	mM	[5]

**pk**	Pyruvate kinase	nefru16bp	1.8	_	[5]
**pk**	Pyruvate kinase	KminPEP	0.08	mM	[5]
**pk**	Pyruvate kinase	KDPPEP	0.58	mM	[5]
**pk**	Pyruvate kinase	KPEP	1.1	mM	[5]
**pk**	Pyruvate kinase	alfaend	1	_	[5]
**pk**	Pyruvate kinase	alfaDP	1	_	[5]
**pk**	Pyruvate kinase	alfaP	1.1	_	[5]
**pk**	Pyruvate kinase	KiPyr	1	mM	
**ppase**	Pyrophosphatase	V	Fitted	$pmol{\cdot}min^{-1}{\cdot}cell^{-1}$	
**ppase**	Pyrophosphatase	KPPi	0.005	mM	[5]
**rpi**	Ribose-5-phosphate isomerase	V	2	$pmol{\cdot}min^{-1}{\cdot}cell^{-1}$	
**rpi**	Ribose-5-phosphate isomerase	Keq	1.20	_	[20]
**rpi**	Ribose-5-phosphate isomerase	KRul5P	0.78	mM	[20]
**rpi**	Ribose-5-phosphate isomerase	KRib5P	2.2	mM	[20]
**rpi**	Ribose-5-phosphate isomerase	KiPGn	6	mM	[20]
**rul5pepi**	Ribulose-5-phosphate 4-epimerase	V	2	$pmol{\cdot}min^{-1}{\cdot}cell^{-1}$	
**rul5pepi**	Ribulose-5-phosphate 4-epimerase	Keq	1.82	_	[20]
**rul5pepi**	Ribulose-5-phosphate 4-epimerase	KRul5P	0.19	mM	[20]
**rul5pepi**	Ribulose-5-phosphate 4-epimerase	KXyl5P	0.5	mM	[20]
**scs**	Succinyl-CoA synthetase	V	Fitted	$pmol{\cdot}min^{-1}{\cdot}cell^{-1}$	
**scs**	Succinyl-CoA synthetase	Keq	1.33333	_	[26]
**scs**	Succinyl-CoA synthetase	KSuCoA	0.086	mM	
**scs**	Succinyl-CoA synthetase	KGDP	0.007	mM	
**scs**	Succinyl-CoA synthetase	KPi	2.26	mM	[48]
**scs**	Succinyl-CoA synthetase	KSuc	0.49	mM	[48]

**scs**	Succinyl-CoA synthetase	KGTP	0.036	mM	[48]
**scs**	Succinyl-CoA synthetase	KCoA	0.036	mM	[48]
**sdh**	Succinate dehydrogenase	V	Fitted	$pmol\cdot min^{-1}\cdot cell^{-1}$	
**sdh**	Succinate dehydrogenase	Keq	1	_	[1]
**sdh**	Succinate dehydrogenase	KiCoQ	0.01	mM	
**sdh**	Succinate dehydrogenase	KSuc	0.03	mM	[49]
**sdh**	Succinate dehydrogenase	KiSuc	0.03	mM	
**sdh**	Succinate dehydrogenase	KFum	0.025	mM	[49]
**sdh**	Succinate dehydrogenase	KiFum	0.025	mM	
**sdh**	Succinate dehydrogenase	KiCoQH	0.2	mM	
**Ta/ta_inv1 /ta_inv2**	Transaldolase / Transaldolase invisible (1) / Transaldolase invisible (2)	e0	Fitted	$pmol\cdot cell^{-1}$	
**Ta/ta_inv1 /ta_inv2**	Transaldolase / Transaldolase invisible (1) / Transaldolase invisible (2)	K1	113.595	min^{-1}	[50]
**Ta/ta_inv1 /ta_inv2**	Transaldolase / Transaldolase invisible (1) / Transaldolase invisible (2)	K2	2887.37	min^{-1}	[50]
**Ta/ta_inv1 /ta_inv2**	Transaldolase / Transaldolase invisible (1) / Transaldolase invisible (2)	K3	5000	min^{-1}	[50]
**Ta/ta_inv1 /ta_inv2**	Transaldolase / Transaldolase invisible (1) / Transaldolase invisible (2)	K4	622860	min^{-1}	[50]
**Ta/ta_inv1 /ta_inv2**	Transaldolase / Transaldolase invisible (1) / Transaldolase invisible (2)	K5	5300	min^{-1}	[50]
**Ta/ta_inv1 /ta_inv2**	Transaldolase / Transaldolase invisible (1) / Transaldolase invisible (2)	K6	5000	min^{-1}	[50]
**Ta/ta_inv1 /ta_inv2**	Transaldolase / Transaldolase invisible (1) / Transaldolase invisible (2)	Kr1	5000	min^{-1}	[50]
**Ta/ta_inv1 /ta_inv2**	Transaldolase / Transaldolase invisible (1) / Transaldolase invisible (2)	Kr2	5300	min^{-1}	[50]

**Ta/ta_inv1 /ta_inv2**	Transaldolase / Transaldolase invisible (1) / Transaldolase invisible (2)	Kr3	6.85627*10^8	min^{-1}	[50]
**Ta/ta_inv1 /ta_inv2**	Transaldolase / Transaldolase invisible (1) / Transaldolase invisible (2)	Kr4	5000	min^{-1}	[50]
**Ta/ta_inv1 /ta_inv2**	Transaldolase / Transaldolase invisible (1) / Transaldolase invisible (2)	Kr5	1770	min^{-1}	[50]
**Ta/ta_inv1 /ta_inv2**	Transaldolase / Transaldolase invisible (1) / Transaldolase invisible (2)	Kr6	0.0622865	min^{-1}	[50]
**tim**	Triose-phosphate isomerase	V	2	$pmol \cdot min^{-1} \cdot cell^{-1}$	
**tim**	Triose-phosphate isomerase	Keq	0.045	_	[1]
**tim**	Triose-phosphate isomerase	KDhaP	0.59	mM	[51]
**tim**	Triose-phosphate isomerase	KGraP	0.4	mM	[51]
**tim**	Triose-phosphate isomerase	Keq	0.045	_	
**tk1/tk2/tk3/tk_inv1/tk_inv2/ tk_inv3**	Transketolase (1) / Transketolase (2) / Transketolase (3) / Transketolase invisible (1) / Transketolase invisible (2) / Transketolase invisible (3)	e0	Fitted	$pmol \cdot cell^{-1}$	
**tk1/tk2/tk3/tk_inv1/tk_inv2/ tk_inv3**	Transketolase (1) / Transketolase (2) / Transketolase (3) / Transketolase invisible (1) / Transketolase invisible (2) / Transketolase invisible (3)	K1	1.07902×10^{7}	min^{-1}	[50]
**tk1/tk2/tk3/tk_inv1/tk_inv2/ tk_inv3**	Transketolase (1) / Transketolase (2) / Transketolase (3) / Transketolase invisible (1) / Transketolase invisible (2) / Transketolase invisible (3)	K2	5300	min^{-1}	[50]
**tk1/tk2/tk3/tk_inv1/tk_inv2/ tk_inv3**	Transketolase (1) / Transketolase (2) / Transketolase (3) / Transketolase invisible (1) / Transketolase invisible (2) / Transketolase invisible (3)	K3	5000	min^{-1}	[50]
**tk1/tk2/tk3/tk_inv1/tk_inv2/ tk_inv3**	Transketolase (1) / Transketolase (2) / Transketolase (3) / Transketolase invisible (1) / Transketolase invisible (2) / Transketolase invisible (3)	K4	11760.9	min^{-1}	[50]

**tk1/tk2/tk3/tk_inv1/tk_inv2/ tk_inv3**	Transketolase (1) / Transketolase (2) / Transketolase (3) / Transketolase invisible (1) / Transketolase invisible (2) / Transketolase invisible (3)	K5	5300	min^{-1}	[50]
**tk1/tk2/tk3/tk_inv1/tk_inv2/ tk_inv3**	Transketolase (1) / Transketolase (2) / Transketolase (3) / Transketolase invisible (1) / Transketolase invisible (2) / Transketolase invisible (3)	K6	5000	min^{-1}	[50]
**tk1/tk2/tk3/tk_inv1/tk_inv2/ tk_inv3**	Transketolase (1) / Transketolase (2) / Transketolase (3) / Transketolase invisible (1) / Transketolase invisible (2) / Transketolase invisible (3)	K7	63384.6	min^{-1}	[50]
**tk1/tk2/tk3/tk_inv1/tk_inv2/ tk_inv3**	Transketolase (1) / Transketolase (2) / Transketolase (3) / Transketolase invisible (1) / Transketolase invisible (2) / Transketolase invisible (3)	K8	98806.8	min^{-1}	[50]
**tk1/tk2/tk3/tk_inv1/tk_inv2/ tk_inv3**	Transketolase (1) / Transketolase (2) / Transketolase (3) / Transketolase invisible (1) / Transketolase invisible (2) / Transketolase invisible (3)	K9	5000	min^{-1}	[50]
**tk1/tk2/tk3/tk_inv1/tk_inv2/ tk_inv3**	Transketolase (1) / Transketolase (2) / Transketolase (3) / Transketolase invisible (1) / Transketolase invisible (2) / Transketolase invisible (3)	Kr1	5000	min^{-1}	[50]
**tk1/tk2/tk3/tk_inv1/tk_inv2/ tk_inv3**	Transketolase (1) / Transketolase (2) / Transketolase (3) / Transketolase invisible (1) / Transketolase invisible (2) / Transketolase invisible (3)	Kr2	5300	min^{-1}	[50]
**tk1/tk2/tk3/tk_inv1/tk_inv2/ tk_inv3**	Transketolase (1) / Transketolase (2) / Transketolase (3) / Transketolase invisible (1) / Transketolase invisible (2) / Transketolase invisible (3)	Kr3	3098.97	min^{-1}	[50]
**tk1/tk2/tk3/tk_inv1/tk_inv2/ tk_inv3**	Transketolase (1) / Transketolase (2) / Transketolase (3) / Transketolase invisible (1) / Transketolase invisible (2) / Transketolase invisible (3)	Kr4	5000	min^{-1}	[50]
**tk1/tk2/tk3/tk_inv1/tk_inv2/ tk_inv3**	Transketolase (1) / Transketolase (2) / Transketolase (3) / Transketolase invisible (1) / Transketolase invisible (2) / Transketolase invisible (3)	Kr5	1770	min^{-1}	[50]

**tk1/tk2/tk3/tk_inv1/tk_inv2/ tk_inv3**	Transketolase (1) / Transketolase (2) / Transketolase (3) / Transketolase invisible (1) / Transketolase invisible (2) / Transketolase invisible (3)	Kr6	5.88567*10^7	min^{-1}	[50]
**tk1/tk2/tk3/tk_inv1/tk_inv2/ tk_inv3**	Transketolase (1) / Transketolase (2) / Transketolase (3) / Transketolase invisible (1) / Transketolase invisible (2) / Transketolase invisible (3)	Kr7	5000	min^{-1}	[50]
**tk1/tk2/tk3/tk_inv1/tk_inv2/ tk_inv3**	Transketolase (1) / Transketolase (2) / Transketolase (3) / Transketolase invisible (1) / Transketolase invisible (2) / Transketolase invisible (3)	Kr8	1.46659*10^6	min^{-1}	[50]
**tk1/tk2/tk3/tk_inv1/tk_inv2/ tk_inv3**	Transketolase (1) / Transketolase (2) / Transketolase (3) / Transketolase invisible (1) / Transketolase invisible (2) / Transketolase invisible (3)	Kr9	36.4257	min^{-1}	[50]
**transa**	Transaminase	V	Fitted	$pmol \cdot min^{-1} \cdot cell^{-1}$	
**transa**	Transaminase	Keq	1	_	
**transa**	Transaminase	KKg	1.25	mM	
**transa**	Transaminase	KGlu	5	mM	
**transa**	Transaminase	KiAsp	2	mM	
**trik**	Triokinase	V	Fitted	$pmol \cdot min^{-1} \cdot cell^{-1}$	
**trik**	Triokinase	KiATP	0.2	mM	
**trik**	Triokinase	KGra	0.012	mM	
**trik**	Triokinase	KiGra	0.012	mM	
**ugt**	UDP-glucuronosyltransferase	V	Fitted	$pmol \cdot min^{-1} \cdot cell^{-1}$	
**ugt**	UDP-glucuronosyltransferase	Keq	0.31	mM	[5]
**ugt**	UDP-glucuronosyltransferase	KUTP	0.563	mM	[5]
**ugt**	UDP-glucuronosyltransferase	KiUTP	0.563	mM	
**ugt**	UDP-glucuronosyltransferase	KGlc1P	0.172	mM	[5]
**ugt**	UDP-glucuronosyltransferase	KiGlc1P	0.172	mM	

**ugt**	UDP-glucuronosyltransferase	KPPi	0.166	mM	[5]
**ugt**	UDP-glucuronosyltransferase	KiPPi	0.166	mM	
**ugt**	UDP-glucuronosyltransferase	KUDPGlc	0.05	mM	[5]
**ugt**	UDP-glucuronosyltransferase	KiUDPGlc	0.05	mM	

References

1. Reich JG, Sel'kov EE. Energy Metabolism of the Cell. London: Academic press INC. (London); 1981.
2. Rakhmanova TI, Popova TN. Regulation of 2-oxoglutarate metabolism in rat liver by NADP-isocitrate dehydrogenase and aspartate aminotransferase. Biochemistry (Mosc). 2006;71(2):211-7. PubMed PMID: 16489927.
3. Guarriero-Bobyleva V, Volpi-Becchi MA, Masini A. Parallel partial purification of cytoplasmic and mitochondrial aconitate hydratases from rat liver. Eur J Biochem. 1973;34(3):455-8. PubMed PMID: 4736701.
4. Tanabe T, Nakanishi S, Hashimoto T, Ogiwara H, Nikawa J, Numa S. Acetyl-CoA carboxylase from rat liver. Methods Enzymol. 1981;71 Pt C:5-16. Epub 1981/01/01. PubMed PMID: 6116163.
5. Konig M, Bulik S, Holzhutter HG. Quantifying the contribution of the liver to glucose homeostasis: a detailed kinetic model of human hepatic glucose metabolism. PLoS Comput Biol. 2012;8(6):e1002577. PubMed PMID: 22761565.
6. Luz CM, Konig I, Schirmer RH, Frank R. Human cytosolic adenylate kinase allelozymes; purification and characterization. Biochim Biophys Acta. 1990;1038(1):80-4.
7. Palmieri F. Mitochondrial carrier proteins. FEBS Lett. 1994;346(1):48-54. PubMed PMID: 8206158.
8. Wells TN, Saxty BA. Redox control of catalysis in ATP-citrate lysate from rat liver. Eur J Biochem. 1992;204(1):249-55. Epub 1992/02/15. PubMed PMID: 1740136.
9. Mueggler PA, Wolfe RG. Malate dehydrogenase. Kinetic studies of substrate activation of supernatant enzyme by L-malate. Biochemistry. 1978;17(22):4615-20. Epub 1978/10/31. PubMed PMID: 215188.
10. McReynolds MS, Kitto GB. Purification and properties of Drosophila malate dehydrogenases. Biochim Biophys Acta. 1970;198(2):165-75.
11. Lin JJ, Yang TH, Wahlstrand BD, Fields PA, Somero GN. Phylogenetic relationships and biochemical properties of the duplicated cytosolic and mitochondrial isoforms of malate dehydrogenase from a teleost fish, Sphyraena idiastes. J Mol Evol. 2002;54(1):107-17.
12. Kurz LC, Shah S, Frieden C, Nakra T, Stein RE, Drysdale GR, et al. Catalytic strategy of citrate synthase: subunit interactions revealed as a consequence of a single amino acid change in the oxaloacetate binding site. Biochemistry. 1995;34(41):13278-88. PubMed PMID: 7577912.
13. Rider CC, Taylor CB. Enolase isoenzymes in rat tissues. Electrophoretic, chromatographic, immunological and kinetic properties. Biochim Biophys Acta. 1974;365(1):285-300. PubMed PMID: 4413246.

14. Rangan VS, Smith S. Alteration of the substrate specificity of the malonyl-CoA/acetyl-CoA:acyl carrier protein S-acyltransferase domain of the multifunctional fatty acid synthase by mutation of a single arginine residue. J Biol Chem. 1997;272(18):11975-8. Epub 1997/05/02. PubMed PMID: 9115261.
15. Taketa K, Pogell BM. Allosteric Inhibition of Rat Liver Fructose 1,6-Diphosphatase by Adenosine 5'-Monophosphate. J Biol Chem. 1965;240:651-62. PubMed PMID: 14275118.
16. Okar DA, Manzano A, Navarro-Sabate A, Riera L, Bartrons R, Lange AJ. PFK-2/FBPase-2: maker and breaker of the essential biofactor fructose-2,6-bisphosphate. Trends Biochem Sci. 2001;26(1):30-5. Epub 2001/02/13. doi: S0968-0004(00)01699-6 [pii]. PubMed PMID: 11165514.
17. Hill RL, Teipel JW. The Enzymes. 3rd ed. Boyer PD, ed, editor1971. 539-71 p.
18. Asipu A, Hayward BE, O'Reilly J, Bonthron DT. Properties of normal and mutant recombinant human ketohexokinases and implications for the pathogenesis of essential fructosuria. Diabetes. 2003;52(9):2426-32. Epub 2003/08/28. PubMed PMID: 12941785.
19. Sestoft L, Fleron P. Determination of the kinetic constants of fructose transport and phosphorylation in the perfused rat liver. Biochim Biophys Acta. 1974;345(1):27-38. Epub 1974/04/12. doi: 0005-2736(74)90242-9 [pii]. PubMed PMID: 4365063.
20. Sabate L, Franco R, Canela EI, Centelles JJ, Cascante M. A model of the pentose phosphate pathway in rat liver cells. Mol Cell Biochem. 1995;142(1):9-17. Epub 1995/01/12. PubMed PMID: 7753046.
21. Lambeir AM, Loiseau AM, Kuntz DA, Vellieux FM, Michels PA, Opperdoes FR. The cytosolic and glycosomal glyceraldehyde-3-phosphate dehydrogenase from Trypanosoma brucei. Kinetic properties and comparison with homologous enzymes. Eur J Biochem. 1991;198(2):429-35. PubMed PMID: 2040304.
22. Vandercammen A, Van Schaftingen E. Competitive inhibition of liver glucokinase by its regulatory protein. Eur J Biochem. 1991;200(2):545-51. Epub 1991/09/01. PubMed PMID: 1889417.
23. Monasterio O, Cardenas ML. Kinetic studies of rat liver hexokinase D ('glucokinase') in non-co-operative conditions show an ordered mechanism with MgADP as the last product to be released. The Biochemical journal. 2003;371(Pt 1):29-38. PubMed PMID: 12513690.
24. Veiga-da-Cunha M, Van Schaftingen E. Identification of fructose 6-phosphate- and fructose 1-phosphate-binding residues in the regulatory protein of glucokinase. J Biol Chem. 2002;277(10):8466-73. Epub 2002/01/05. doi: 10.1074/jbc.M105984200

M105984200 [pii]. PubMed PMID: 11756407.
25. Thorens B, Mueckler M. Glucose transporters in the 21st Century. Am J Physiol Endocrinol Metab. 2009;298(2):E141-5. PubMed PMID: 20009031.
26. Goldberg RN, Tewari YB, Bhat TN. Thermodynamics of enzyme-catalyzed reactions--a database for quantitative biochemistry. Bioinformatics. 2004;20(16):2874-7. Epub 004 May 14.
27. Ostro MJ, Fondy TP. Isolation and characterization of multiple molecular forms of cytosolic NAD-linked glycerol-3-phosphate dehydrogenase from normal and neoplastic rabbit tissues. J Biol Chem. 1977;252(15):5575-83. Epub 1977/08/10. PubMed PMID: 195955.
28. Dawson AP, Thorne CJ. L-3-glycerophosphate dehydrogenase from pig brain mitochondria. Methods Enzymol. 1975;41:254-9. Epub 1975/01/01. doi: S0076-6879(75)41058-8 [pii]. PubMed PMID: 1128264.

29. Kanno H, Fujii H, Miwa S. Expression and enzymatic characterization of human glucose phosphate isomerase (GPI) variants accounting for GPI deficiency. Blood Cells Mol Dis. 1998;24(1):54-61. PubMed PMID: 9616041.
30. Bzymek KP, Colman RF. Role of alpha-Asp181, beta-Asp192, and gamma-Asp190 in the distinctive subunits of human NAD-specific isocitrate dehydrogenase. Biochemistry. 2007;46(18):5391-7. PubMed PMID: 17432878.
31. Sheu KF, Blass JP. The alpha-ketoglutarate dehydrogenase complex. Ann N Y Acad Sci. 1999;893:61-78. Epub 2000/02/15. PubMed PMID: 10672230.
32. Wang YC, Wang ST, Li C, Liu WH, Chen PR, Chen LY, et al. The role of N286 and D320 in the reaction mechanism of human dihydrolipoamide dehydrogenase (E3) center domain. J Biomed Sci. 2007;14(2):203-10. PubMed PMID: 17171578.
33. Koike K, Suematsu T, Ehara M. Cloning, overexpression and mutagenesis of cDNA encoding dihydrolipoamide succinyltransferase component of the porcine 2-oxoglutarate dehydrogenase complex. Eur J Biochem. 2000;267(10):3005-16. PubMed PMID: 10806400.
34. Edlund GL, Halestrap AP. The kinetics of transport of lactate and pyruvate into rat hepatocytes. Evidence for the presence of a specific carrier similar to that in erythrocytes. The Biochemical journal. 1988;249(1):117-26. Epub 1988/01/01. PubMed PMID: 3342001; PubMed Central PMCID: PMC1148674.
35. *Eichner RD*. l(+)-Lactate dehydrogenase from Homarus americanus. Methods in Enzymology. 1982;89:359–62.
36. Nakae Y, Stoward PJ. Kinetic parameters of lactate dehydrogenase in liver and gastrocnemius determined by three quantitative histochemical methods. J Histochem Cytochem. 1997;45(10):1427-31. Epub 1997/10/06. PubMed PMID: 9313804.
37. Pettit SM, Nealon DA, Henderson AR. Purification of lactate dehydrogenase isoenzyme-5 from human liver. Clin Chem. 1981;27(1):88-93. Epub 1981/01/01. PubMed PMID: 7449128.
38. Zelewski M, Swierczynski J. Malic enzyme in human liver. Intracellular distribution, purification and properties of cytosolic isozyme. Eur J Biochem. 1991;201(2):339-45. Epub 1991/10/15. PubMed PMID: 1935931.
39. Hung HC, Kuo MW, Chang GG, Liu GY. Characterization of the functional role of allosteric site residue Asp102 in the regulatory mechanism of human mitochondrial NAD(P)+-dependent malate dehydrogenase (malic enzyme). The Biochemical journal. 2005;392(Pt 1):39-45. PubMed PMID: 15989682.
40. Jitrapakdee S, Walker ME, Wallace JC. Functional expression, purification, and characterization of recombinant human pyruvate carboxylase. Biochem Biophys Res Commun. 1999;266(2):512-7.
41. Warren GB, Tipton KF. Pig liver pyruvate carboxylase. Purification, properties and cation specificity. The Biochemical journal. 1974;139(2):297-310. PubMed PMID: 4447611.
42. Scrutton MC, Utter MF. Pyruvate carboxylase. IX. Some properties of the activation by certain acyl derivatives of coenzyme A. J Biol Chem. 1967;242(8):1723-35. Epub 1967/04/25. PubMed PMID: 6024765.
43. Johnson TA, Holyoak T. Increasing the conformational entropy of the Omega-loop lid domain in phosphoenolpyruvate carboxykinase impairs catalysis and decreases catalytic fidelity. Biochemistry. 49(25):5176-87.
44. Laloux M, Van Schaftingen E, Francois J, Hers HG. Phosphate dependency of phosphofructokinase 2. Eur J Biochem. 1985;148(1):155-9. Epub 1985/04/01. PubMed PMID: 2983989.

45. Szabo J, Varga A, Flachner B, Konarev PV, Svergun DI, Zavodszky P, et al. Communication between the nucleotide site and the main molecular hinge of 3-phosphoglycerate kinase. Biochemistry. 2008;47(26):6735-44. PubMed PMID: 18540639.
46. Bartrons R, Carreras J. Purification and characterization of phosphoglycerate mutase isozymes from pig heart. Biochim Biophys Acta. 1982;708(2):167-77. PubMed PMID: 6293580.
47. Yamada K, Noguchi T. Nutrient and hormonal regulation of pyruvate kinase gene expression. The Biochemical journal. 1999;337 (Pt 1):1-11. Epub 1998/12/17. PubMed PMID: 9854017; PubMed Central PMCID: PMC1219928.
48. Johnson JD, Muhonen WW, Lambeth DO. Characterization of the ATP- and GTP-specific succinyl-CoA synthetases in pigeon. The enzymes incorporate the same alpha-subunit. J Biol Chem. 1998;273(42):27573-9. PubMed PMID: 9765290.
49. Grivennikova VG, Gavrikova EV, Timoshin AA, Vinogradov AD. Fumarate reductase activity of bovine heart succinate-ubiquinone reductase. New assay system and overall properties of the reaction. Biochim Biophys Acta. 1993;1140(3):282-92. Epub 1993/01/08. PubMed PMID: 8417779.
50. Selivanov VA, Meshalkina LE, Solovjeva ON, Kuchel PW, Ramos-Montoya A, Kochetov GA, et al. Rapid simulation and analysis of isotopomer distributions using constraints based on enzyme mechanisms: an example from HT29 cancer cells. Bioinformatics. 2005;21(17):3558-64. doi: 10.1093/bioinformatics/bti573.
51. Snyder R, Lee EW. Triosephosphate isomerase from human and horse liver. Methods Enzymol. 1975;41:430-4. PubMed PMID: 1168838.
