# Supplementary material for: HepatoDyn: A Dynamic Model of Hepatocyte Metabolism That Integrates 13C Isotopomer Data
Source: PLoS Comput Biol. 2016 Apr 28;12(4):e1004899. doi: 10.1371/journal.pcbi.1004899 (PMC4849781; doi:10.1371/journal.pcbi.1004899)
Supplement: S5 Table — This table describes the parameters representing enzyme activities that are fitted as a group, indicating the relative value of the enzyme activities associated to each group. (PDF) [file pcbi.1004899.s014.pdf]

S5 Table: Parameter groups. This table describes the parameters representing enzyme activities that are fitted as a group, indicating the relative value of the enzyme activities associated to each group.

Parameter group	Reaction id	Reaction	Parameter	Relative value
Energy metabolism	atpmtrans	Mitochondrial ATP/ADP carrier	V	1
	coqhoxi	Ubiquinol Oxidase	K	100
	nadhdh	NADH dehydrogenase	K	100
	pimtr	Phosphate mitochondrial carrier	V	1
Fatty acid synthesis	acoacar	Acetyl-CoA carboxylase	V	1
	citly	Citrate lyase	V	2
	citmtr	Citrate carrier	V	4
	fasyn	Fatty acid synthesis	V	0.2
	malic	Malic enzyme	V	2
Fructose phosphorylation	fruhk	Fructokinase	V	1
	trik	Triokinase	V	2
Gluconeogenesis	dic	Dicarboxylate Carrier	V	1
	pc	Pyruvate Carboxylase	V	1
	pepck	Phosphoenolpyruvate carboxykinase	V	1
Glutamate production	gluupt	Glutamate carrier	V	1
	transa	Transaminase	V	10
Glycerol phosphate shuttle	glyc3pdh	Glycerol-3-phosphate dehydrogenase (NAD)	V	1
	glyc3pmdh	Glycerol-3-phosphate dehydrogenase (Ubiquinone)	V	100
Glycogen synthesis	gs	Glycogen synthase	V	1
	ppase	Pyrophosphatase	V	5
	ugt	UDP-glucuronosyltransferase	V	2
Lactate	lacupt	Lactate carrier / Pyruvate extracellular carrier	V	1

production				
	ldh	Lactate dehydrogenase	V	2
Lower Krebs cycle	fh	Fumarate Hydratase	V	10
	kdh	α-Ketoglutarate dehydrogenase	V	1
	mmdh	Malate dehydrogenase (Mitochondrial)	V	10
	scs	Succinyl-CoA synthetase	V	2
	sdh	Succinate dehydrogenase	V	2
Malate aspartate shuttle	aatc	Aspartate aminotransferase (Cytosolic)	V	10
	atm	Aspartate aminotransferase (Mitochondrial)	V	10
	cmdh	Malate dehydrogenase (Cytosolic)	V	10
	malkgmtrans	α-Ketoglutarate/Malate carrier	V	1
	aspglumtrans	Aspartate/Glutamate carrier	V	1
Pentose phosphate pathway	g6pdh	Glucose-6-Phosphate deshydrogenase	V	1
	pgndh	Phosphogluconate dehydrogenase	V	1
	ta/ ta_inv1/ta_inv2	Transaldolase / Transaldolase invisible(1) / Transaldolase invisible (2)	E0	10
	tk1/tk2/tk3/tk_inv1/tk_inv2/tk_inv3	Transketolase (1) / Transketolase (2) / Transketolase (3) / Transketolase invisible (1) / Transketolase invisible (2) / Transketolase invisible (3)	E0	10
