# Supplementary material for: HepatoDyn: A Dynamic Model of Hepatocyte Metabolism That Integrates 13C Isotopomer Data
Source: PLoS Comput Biol. 2016 Apr 28;12(4):e1004899. doi: 10.1371/journal.pcbi.1004899 (PMC4849781; doi:10.1371/journal.pcbi.1004899)
Supplement: S6 Table — This table describes the predicted values for all parameters that have been fitted to the experimental data. In addition to the best fit value, the confidence intervals for 95% confidence according to identifiability analysis are also shown. (PDF) [file pcbi.1004899.s015.pdf]

**S6 Table: Parameters fitted to experimental conditions.** This table describes the predicted values for all parameters that have been fitted to the experimental data. In addition to the best fit value, the confidence intervals for 95% confidence according to identifiability analysis are also shown.

| Reaction(s) id | Reaction(s) | Parameter | Best fit | 95% Confidence interval | Units | Parameter group |
|---|---|---|---|---|---|---|
| **atpmtrans** | Mitochondrial ATP/ADP carrier | V | 3.3878 | (1.5804, 9.6657) | $pmol \cdot min^{-1} \cdot cell^{-1}$ | Energy metabolism |
| **coqhoxi** | Ubiquinol Oxidase | K | 338.775 | (158.0410, 966.5670) | $l^5 \cdot mmol^{-4} \cdot cell^{-1}$ | |
| **nadhdh** | NADH dehydrogenase | K | 338.775 | (158.0410, 966.5670) | $l^4 \cdot mmol^{-3} \cdot cell^{-1}$ | |
| **pimtr** | Phosphate mitochondrial carrier | V | 3.3878 | (1.5804, 9.6657 | $pmol \cdot min^{-1} \cdot cell^{-1}$ | |
| **acoacar** | Acetyl-CoA carboxylase | V | 0.0003 | (0.0000, 0.0153 | $pmol \cdot min^{-1} \cdot cell^{-1}$ | Fatty acid synthesis |
| **citly** | Citrate lyase | V | 0.0007 | (0.0000, 0.0307 | $pmol \cdot min^{-1} \cdot cell^{-1}$ | |
| **citmtr** | Citrate carrier | V | 0.0014 | (0.0000, 0.0614 | $pmol \cdot min^{-1} \cdot cell^{-1}$ | |
| **fasyn** | Fatty acid synthesis | V | 0.0001 | (0.0000, 0.0031 | $pmol \cdot min^{-1} \cdot cell^{-1}$ | |
| **malic** | Malic enzyme | V | 0.0007 | (0.0000, 0.0307 | $pmol \cdot min^{-1} \cdot cell^{-1}$ | |
| **fruhk** | Fructokinase | V | 0.0949 | (0.0536, 0.1682 | $pmol \cdot min^{-1} \cdot cell^{-1}$ | Fructose phosphorylation |
| **trik** | Triokinase | V | 0.1899 | (0.1072, 0.3364 | $pmol \cdot min^{-1} \cdot cell^{-1}$ | |
| **dic** | Dicarboxylate Carrier | V | 0.1835 | (0.0531, 3.2017 | $pmol \cdot min^{-1} \cdot cell^{-1}$ | Gluconeogenesis |
| **pc** | Pyruvate Carboxylase | V | 0.1835 | (0.0531, 3.2017 | $pmol \cdot min^{-1} \cdot cell^{-1}$ | |
| **pepck** | Phosphoenolpyruvate carboxykinase | V | 0.1835 | (0.0531, 3.2017 | $pmol \cdot min^{-1} \cdot cell^{-1}$ | |

| | | | | | | |
|---|---|---|---|---|---|---|
| **glutr** | Glutamate carrier | V | 0.0016 | (0.0006, 0.0073 | pmol·min$^{-1}$·cell$^{-1}$ | Glutamate production |
| **transa** | Transaminase | V | 0.0159 | (0.0056, 0.0729 | pmol·min$^{-1}$·cell$^{-1}$ | |
| **glyc3pdh** | Glycerol-3-phosphate dehydrogenase (NAD) | V | 0.0000 | (0.0000, 0.0129 | pmol·min$^{-1}$·cell$^{-1}$ | Glycerol phosphate shuttle |
| **glyc3pmdh** | Glycerol-3-phosphate dehydrogenase (Ubiquinone) | V | 0.0005 | (0.0000, 1.2935 | pmol·min$^{-1}$·cell$^{-1}$ | |
| **gs** | Glycogen synthase | V | 0.0490 | (0.0189, 3.9644 | pmol·min$^{-1}$·cell$^{-1}$ | Glycogen synthesis |
| **ppase** | Pyrophosphatase | V | 0.2448 | (0.0944, 19.8221 | pmol·min$^{-1}$·cell$^{-1}$ | |
| **ugtv** | UDP-glucuronosyltransferase | V | 0.0979 | (0.0378, 7.9288 | pmol·min$^{-1}$·cell$^{-1}$ | |
| **lactr** | Lactate carrier / Pyruvate extracellular carrier | V | 0.1632 | (0.0201, ∞ | pmol·min$^{-1}$·cell$^{-1}$ | Lactate production |
| **ldh** | Lactate dehydrogenase | V | 0.3265 | (0.0401, ∞ | pmol·min$^{-1}$·cell$^{-1}$ | |
| **fh** | Fumarate Hydratase | V | 3.1493 | (0.9122, 13.1553 | pmol·min$^{-1}$·cell$^{-1}$ | Lower Krebs cycle |
| **kdh** | α-Ketoglutarate dehydrogenase | V | 0.3149 | (0.0912, 1.3155 | pmol·min$^{-1}$·cell$^{-1}$ | |
| **mmdh** | Malate dehydrogenase (Mitochondrial) | V | 3.1493 | (0.9122, 13.1553 | pmol·min$^{-1}$·cell$^{-1}$ | |

| | | | | | | |
|---|---|---|---|---|---|---|
| **scs** | Succinyl-CoA synthetase | V | 0.6299 | (0.1824, 2.6311 | pmol·min$^{-1}$·cell$^{-1}$ | |
| **sdh** | Succinate dehydrogenase | V | 0.6299 | (0.1824, 2.6311) | pmol·min$^{-1}$·cell$^{-1}$ | |
| **aatc** | Aspartate aminotransferase (Cytosolic) | V | 32.0705 | (6.9795, ∞) | pmol·min$^{-1}$·cell$^{-1}$ | Malate aspartate shuttle |
| **aatm** | Aspartate aminotransferase (Mitochondrial) | V | 32.0705 | (6.9795, ∞) | pmol·min$^{-1}$·cell$^{-1}$ | |
| **cmdh** | Malate dehydrogenase (Cytosolic) | V | 32.0705 | (6.9795, ∞) | pmol·min$^{-1}$·cell$^{-1}$ | |
| **malkgmtrans** | α-Ketoglutarate/Malate carrier | V | 3.2071 | (0.6979, ∞) | pmol·min$^{-1}$·cell$^{-1}$ | |
| **aspglumtrans** | Aspartate/Glutamate carrier | V | 3.2071 | (0.6979, ∞) | pmol·min$^{-1}$·cell$^{-1}$ | |
| **g6pdh** | Glucose-6-Phosphate deshydrogenase | V | 0.0386 | (0.0000, 0.3803) | pmol·min$^{-1}$·cell$^{-1}$ | Pentose phosphate pathway |
| **pgndh** | Phosphogluconate dehydrogenase | V | 0.0386 | (0.0000, 0.3803) | pmol·min$^{-1}$·cell$^{-1}$ | |
| **ta / ta_inv1/ ta_inv2** | Transaldolase / Transaldolase invisible(1) / Transaldolase invisible (2) | E0 | 3.8609 | (0.0000, 38.0290) | pmol·min$^{-1}$·cell$^{-1}$ | |
| **tk1 / tk2 / tk3 / tk_inv1 / tk_inv2 / tk_inv3** | Transketolase (1) / Transketolase (2) / Transketolase (3) / Transketolase | E0 | 3.8609 | (0.0000, 38.0290) | pmol·min$^{-1}$·cell$^{-1}$ | |

| | | | | | | |
|---|---|---|---|---|---|---|
| | invisible (1) / Transketolase invisible (2) / Transketolase invisible (3) | | | | | |
| **aldo1 / aldo2 / aldo3 / aldo_inv1 / aldo_inv2** | Aldolase (1) / Aldolase (2) / Aldolase (3) / Aldolase invisible (1) / Aldolase invisible (2) | E0 | 1.2463 | (0.1046, ∞) | pmol·min$^{-1}$·cell$^{-1}$ | - |
| **atpase** | Atpase | V | 0.0163 | (0.0000, 0.1770) | pmol·min$^{-1}$·cell$^{-1}$ | - |
| **box** | β-Oxidation | V | 0.0013 | (0.0007, 0.0023) | pmol·min$^{-1}$·cell$^{-1}$ | - |
| **cs** | Citrate synthase | V | 0.0673 | (0.0068, ∞) | pmol·min$^{-1}$·cell$^{-1}$ | - |
| **fbpasela** | Fructose 1,6-bisphosphatase (Pool A) | V | 0.5172 | (0.1039, 1.3415) | pmol·min$^{-1}$·cell$^{-1}$ | - |
| **fbpaselb** | Fructose 1,6-bisphosphatase (Pool B) | V | 0.0052 | (0.0012, 0.0347) | pmol·min$^{-1}$·cell$^{-1}$ | - |
| **frutr** | Fructose carrier | V | 0.8393 | (0.5211, 1.3517) | pmol·min$^{-1}$·cell$^{-1}$ | - |
| **g6pasea** | Glucose-6-Phosphatase (Pool A) | V | 0.0578 | (0.0176, 0.3988) | pmol·min$^{-1}$·cell$^{-1}$ | - |
| **g6paseb** | Glucose-6-Phosphatase (Pool B) | V | 0.0383 | (0.0012, 5.4418) | pmol·min$^{-1}$·cell$^{-1}$ | - |
| **gka** | Glucokinase (Pool A) | V | 0.0131 | (0.0047, 0.4041) | pmol·min$^{-1}$·cell$^{-1}$ | - |

| | | | | | | |
|---|---|---|---|---|---|---|
| **gkb** | Glucokinase (Pool B) | V | 0.0089 | (0.0028, 0.0448) | $pmol \cdot min^{-1} \cdot cell^{-1}$ | - |
| **gp** | Glycogen Phosphorylase | V | 0.0004 | (0.0000, 169.085 | $pmol \cdot min^{-1} \cdot cell^{-1}$ | - |
| **idh** | Isocitrate dehydrogenase | V | 0.0098 | (0.0061, 0.0209) | $pmol \cdot min^{-1} \cdot cell^{-1}$ | - |
| **pdh** | Pyruvate dehydrogenase | V | 0.0024 | (0.0013, 0.0109) | $pmol \cdot min^{-1} \cdot cell^{-1}$ | - |
| **pfkla** | Phosphofructokinase 1(Pool A) | V | 0.0126 | (0.0004, 0.2666) | $pmol \cdot min^{-1} \cdot cell^{-1}$ | - |
| **pfklb** | Phosphofructokinase 1(Pool B) | V | 0.0000 | (0.0000, 0.0144) | $pmol \cdot min^{-1} \cdot cell^{-1}$ | - |
| **pitr** | Phosphate carrier | V | 0.0000 | (0.0000, 0.0038) | $pmol \cdot min^{-1} \cdot cell^{-1}$ | - |
| **pk** | Pyruvate kinase | V | 0.0601 | (0.0411, 2.0439) | $pmol \cdot min^{-1} \cdot cell^{-1}$ | - |
