# Supplementary material for: HepatoDyn: A Dynamic Model of Hepatocyte Metabolism That Integrates 13C Isotopomer Data
Source: PLoS Comput Biol. 2016 Apr 28;12(4):e1004899. doi: 10.1371/journal.pcbi.1004899 (PMC4849781; doi:10.1371/journal.pcbi.1004899)
Supplement: S1 Text — This text describes the kinetic laws used for the aldolase reactions and how they have been constructed. (PDF) [file pcbi.1004899.s016.pdf]

## S1 Text

**Kinetic laws used for the reactions associated with aldolase activity**. This text describes the kinetic laws used for the aldolase reactions and how they have been constructed.

Firstly, the laws for rate of consumption and production of fructose-1-6 bisphosphate (FBP), fructose-1-phosphate (Fru1P), dihydroxyacetone phosphate (DHAP), glyceraldehyde 3-phosphate (GraP), and glyceraldehyde (Gra) were derived following the King-Altman method [1]. The first step of this method is to represent the mechanism in a closed scheme showing all enzyme species (free enzyme and enzyme complexes) and possible reactions. Specifically for the aldolase enzyme, the enzyme species are free Enzyme (E), enzyme complexed with fructose-1-6 bisphosphate (E-FBP), enzyme complexed with fructose-1-phosphate (E-Fr1P) and enzyme complexed with dihydroxyacetone phosphate (E-DHAP). The mechanism can be written as follows:

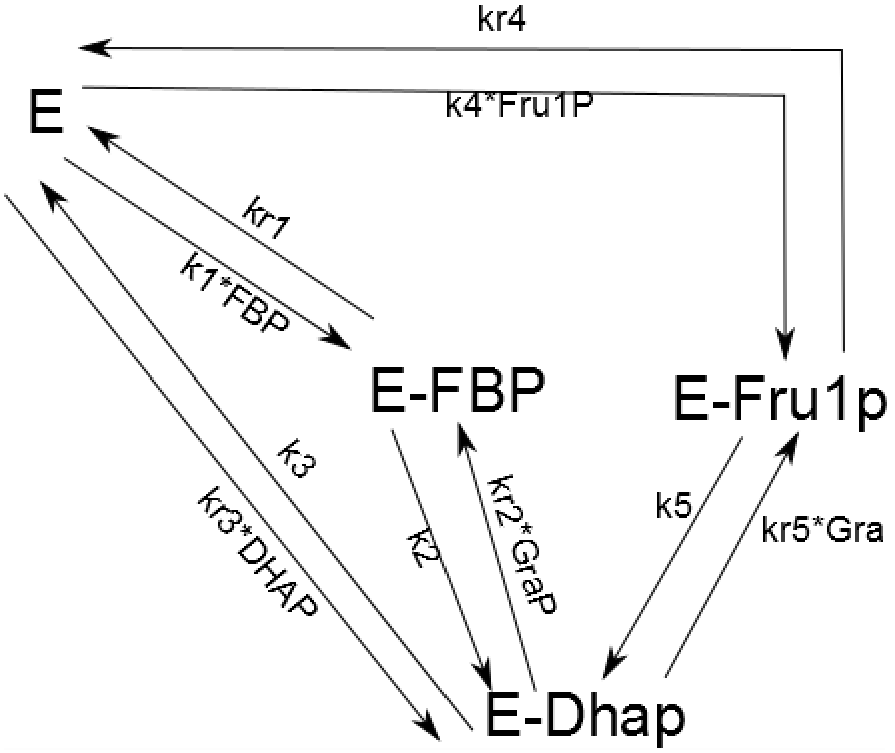


Where rate constants are accounting for the forward (k1,k2,k3,k4,k5) and reverse reactions (kr1,kr2,kr3,kr4,kr5). From this scheme the relative concentrations of each enzyme species are derived:

$$\frac{[E]}{e0} = (k2\,k3\,k5 + k3\,k5\,kr1 + grap\,k5\,kr1\,kr2 + k2\,k3\,kr4 + k3\,kr1\,kr4 + grap\,kr1\,kr2\,kr4 + gra\,k2\,kr4\,kr5 + gra\,kr1\,kr4\,kr5)/Denom$$

$$\frac{[E\ FBP]}{e0} = (fbp\ k1\ k3\ k5\ +\ fbp\ grap\ k1\ k5\ kr2\ +\ fru1p\ grap\ k4\ k5\ kr2 \\ +\ dhap\ grap\ k5\ kr2\ kr3\ +\ fbp\ k1\ k3\ kr4\ +\ fbp\ grap\ k1\ kr2\ kr4 \\ +\ dhap\ grap\ kr2\ kr3\ kr4\ +\ fbp\ gra\ k1\ kr4\ kr5)/Denom$$

$$\frac{[E\ DHAP]}{e0} = (fbp\ k1\ k2\ k5 + fru1p\ k2\ k4\ k5 + fru1p\ k4\ k5\ kr1 + dhap\ k2\ k5\ kr3 \\ + dhap\ k5\ kr1\ kr3 + fbp\ k1\ k2\ kr4 + dhap\ k2\ kr3\ kr4 \\ + dhap\ kr1\ kr3\ kr4)/Denom$$

$$\frac{[E\ Fr1P]}{e0} = (fru1p\ k2\ k3\ k4\ +\ fru1p\ k3\ k4\ kr1\ +\ fru1p\ grap\ k4\ kr1\ kr2 \\ +\ fbp\ gra\ k1\ k2\ kr5\ +\ fru1p\ gra\ k2\ k4\ kr5 \\ +\ fru1p\ gra\ k4\ kr1\ kr5\ +\ dhap\ gra\ k2\ kr3\ kr5 \\ +\ dhap\ gra\ kr1\ kr3\ kr5)/Denom$$

Where Denom is:

$$Denom = k2\ k3\ k5\ +\ k3\ k5\ kr1\ +\ grap\ k5\ kr1\ kr2\ +\ k2\ k3\ kr4\ +\ k3\ kr1\ kr4 \\ +\ grap\ kr1\ kr2\ kr4\ +\ gra\ k2\ kr4\ kr5\ +\ gra\ kr1\ kr4\ kr5 \\ + fbp\ k1\ k3\ k5\ +\ fbp\ grap\ k1\ k5\ kr2\ +\ fru1p\ grap\ k4\ k5\ kr2 \\ +\ dhap\ grap\ k5\ kr2\ kr3\ +\ fbp\ k1\ k3\ kr4\ +\ fbp\ grap\ k1\ kr2\ kr4 \\ +\ dhap\ grap\ kr2\ kr3\ kr4\ +\ fbp\ gra\ k1\ kr4\ kr5 +\ fbp\ k1\ k2\ k5 \\ + fru1p\ k2\ k4\ k5 + fru1p\ k4\ k5\ kr1 + dhap\ k2\ k5\ kr3 \\ + dhap\ k5\ kr1\ kr3 + fbp\ k1\ k2\ kr4 + dhap\ k2\ kr3\ kr4 \\ + dhap\ kr1\ kr3\ kr4 + fru1p\ k2\ k3\ k4\ +\ fru1p\ k3\ k4\ kr1 \\ +\ fru1p\ grap\ k4\ kr1\ kr2\ +\ fbp\ gra\ k1\ k2\ kr5\ +\ fru1p\ gra\ k2\ k4\ kr5 \\ +\ fru1p\ gra\ k4\ kr1\ kr5\ +\ dhap\ gra\ k2\ kr3\ kr5 \\ +\ dhap\ gra\ kr1\ kr3\ kr5$$

Then, knowing the relative concentration of each enzyme species allows the construction of a kinetic law for the forward (v1,v2,v3,v4,v5) or reverse (vr1,vr2,vr3,vr4,vr5) reaction accounting for the consumption or production of each metabolite, specifically:

Fru16bP Consumption (v1):

$$v1 = e0 * \frac{k1 * fbp * [E]}{Denom} \\ = k1 * fbp * (k2\ k3\ k5\ +\ k3\ k5\ kr1\ +\ grap\ k5\ kr1\ kr2\ +\ k2\ k3\ kr4 \\ +\ k3\ kr1\ kr4\ +\ grap\ kr1\ kr2\ kr4\ +\ gra\ k2\ kr4\ kr5 \\ +\ gra\ kr1\ kr4\ kr5)/Denom$$

Fru16bP Production (vr1):

$$vr1 = e0 * \frac{kr1 * [Efbp]}{Denom}$$
$$= kr1 * (fbp\ k1\ k3\ k5\ +\ fbp\ grap\ k1\ k5\ kr2\ +\ fru1p\ grap\ k4\ k5\ kr2 + dhap\ grap\ k5\ kr2\ kr3\ +\ fbp\ k1\ k3\ kr4\ +\ fbp\ grap\ k1\ kr2\ kr4 + dhap\ grap\ kr2\ kr3\ kr4\ +\ fbp\ gra\ k1\ kr4\ kr5)/Denom$$

GraP Production (v2):

$$v2 = \frac{e0 * k2 * [Efbp]}{Denom}$$
$$= e0 * k2 * (fbp\ k1\ k3\ k5\ +\ fbp\ grap\ k1\ k5\ kr2\ +\ fru1p\ grap\ k4\ k5\ kr2 + dhap\ grap\ k5\ kr2\ kr3\ +\ fbp\ k1\ k3\ kr4\ +\ fbp\ grap\ k1\ kr2\ kr4 + dhap\ grap\ kr2\ kr3\ kr4\ +\ fbp\ gra\ k1\ kr4\ kr5)/Denom$$

GraP Consumption (vr2):

$$vr2 = \frac{e0 * kr2 * [E\ DHAP] * grap}{Denom}$$
$$= e0 * kr2 * grap * (fbp\ k1\ k2\ k5 + fru1p\ k2\ k4\ k5 + fru1p\ k4\ k5\ kr1 + dhap\ k2\ k5\ kr3 + dhap\ k5\ kr1\ kr3 + fbp\ k1\ k2\ kr4 + dhap\ k2\ kr3\ kr4 + dhap\ kr1\ kr3\ kr4)/Denom$$

DHAP Production (v3):

$$v3 = \frac{e0 * k3 * [E\ DHAP]}{Denom}$$
$$= e0 * k3 * (fbp\ k1\ k2\ k5 + fru1p\ k2\ k4\ k5 + fru1p\ k4\ k5\ kr1 + dhap\ k2\ k5\ kr3 + dhap\ k5\ kr1\ kr3 + fbp\ k1\ k2\ kr4 + dhap\ k2\ kr3\ kr4 + dhap\ kr1\ kr3\ kr4)/Denom$$

Dhap consumption (vr3):

$$vr3 = \frac{e0 * kr3 * dhap[E]}{Denom}$$
$$= e0 * kr3 * dhap(k2\ k3\ k5\ +\ k3\ k5\ kr1\ +\ grap\ k5\ kr1\ kr2 + k2\ k3\ kr4\ +\ k3\ kr1\ kr4\ +\ grap\ kr1\ kr2\ kr4\ +\ gra\ k2\ kr4\ kr5 + gra\ kr1\ kr4\ kr5)/Denom$$

Fru1P Consumption (v4):

$$v4\ =\ e0 * \frac{k4 * [E] * fru1p}{Denom}$$
$$= k4 * fru1p(k2\ k3\ k5\ +\ k3\ k5\ kr1\ +\ grap\ k5\ kr1\ kr2\ +\ k2\ k3\ kr4 + k3\ kr1\ kr4\ +\ grap\ kr1\ kr2\ kr4\ +\ gra\ k2\ kr4\ kr5 + gra\ kr1\ kr4\ kr5)/Denom$$

Fr1P Production (vr4):

$$vr4 = e0 * \frac{kr4 * [Efru1p]}{Denom}$$
$$= kr4 * (fru1p\ k2\ k3\ k4\ +\ fru1p\ k3\ k4\ kr1 + fru1p\ grap\ k4\ kr1\ kr2\ +\ fbp\ gra\ k1\ k2\ kr5 + fru1p\ gra\ k2\ k4\ kr5\ +\ fru1p\ gra\ k4\ kr1\ kr5 + dhap\ gra\ k2\ kr3\ kr5\ +\ dhap\ gra\ kr1\ kr3\ kr5)/Denom$$

Gra Production (v5):

$$v5 = \frac{e0 * k5 * [Efru1p]}{Denom}$$
$$= e0 * k5 * (\mathrm{fru1p\ k2\ k3\ k4\ +\ fru1p\ k3\ k4\ kr1 + fru1p\ grap\ k4\ kr1\ kr2\ +\ fbp\ gra\ k1\ k2\ kr5 + fru1p\ gra\ k2\ k4\ kr5\ +\ fru1p\ gra\ k4\ kr1\ kr5 + dhap\ gra\ k2\ kr3\ kr5\ +\ dhap\ gra\ kr1\ kr3\ kr5})/\mathrm{Denom}$$

Gra Consumption (vr5):

$$vr5 = \frac{e0 * kr5 * [EDhap] * Gra}{Denom}$$
$$= e0 * kr5 * Gra * (fbp\ k1\ k2\ k5 + fru1p\ k2\ k4\ k5 + fru1p\ k4\ k5\ kr1 + dhap\ k2\ k5\ kr3 + dhap\ k5\ kr1\ kr3 + fbp\ k1\ k2\ kr4 + dhap\ k2\ kr3\ kr4 + dhap\ kr1\ kr3\ kr4)/Denom$$

To obtain the kinetic rate equations that describe the fluxes that propagate labels through the aldolase reaction from the previously derived reaction rates, we followed a previously described method [2]. Firstly, a system of equations is written defining the following fractions: fraction of enzyme-complexed DHAP originated from FBP (Pdhapfromfbp), enzyme-complexed DHAP originated from Fr1p (Pdhapfromfru1p), enzyme-complexed DHAP originated from DHAP (Pdhapfromdhap), enzyme-complexed Fr1P originated from FBP (Fr1ppfromfbp), enzyme-complexed Fr1p originated from Fr1p (fr1pfromfru1p), enzyme-complexed Fr1p originated from DHAP (Fr1pfromdhap), enzyme-complexed FBP originated from FBP (Pfbpfromfbp), enzyme-complexed FBP originated from dhap (Pfbpfromfru1p), and enzyme-complexed FBP originated from DHAP (Pfbpfromdhap).

$$Pdhapfromfbp = (v2 * Pfbpfromfbp + v5 * Pfr1pfromfbp)/(v5 + v2 + vr3)$$

$$Pdhapfromfru1p = (v5 * Pfru1pfromfru1p + v2 * Pfbpfromfru1p)/(v5 + v2 + vr3)$$

$$Pfbpfromfbp = (vr2 * Pdhapfromfbp + v1)/(v1 + vr2)$$

$$Pfru1pfromfru1p = (v4 + vr5 * Pdhapfromfru1p)/(v4 + vr5)$$

$$Pfbpfromfru1p = vr2 * Pdhapfromfru1p/(v1 + vr2)$$

$$Pfr1pfromfbp = vr5 * Pdhapfromfbp/(v4 + vr5)$$

$$Pfbpfromdhap = Pdhapfromdhap * vr2/(v1 + vr2)$$

$$Pfr1pfromdhap = Pdhapfromdhap * \frac{vr5}{v4 + vr5}$$

$$1 = \mathrm{Pdhapfromfbp} + \mathrm{Pdhapfromfru1p} + \mathrm{Pdhapfromdhap}$$

$$1 = \mathrm{Pfbpfromfbp} + \mathrm{Pfbpfromfru1p} + \mathrm{Pfbpfromdhap}$$

$$1 = \mathrm{Pfru1pfromfru1p} + \mathrm{Pfr1pfromfbp} + \mathrm{Pfr1pfromdhap}$$

Solving the system gives:

$$\mathrm{Pdhapfromfbp} = \frac{\mathrm{v1v2(v4 + vr5)}}{\mathrm{v1v2v4 + v1v4v5 + v4v5vr2 + v1v4vr3 + v4vr2vr3 + v1v2vr5 + v1vr3vr5 + vr2vr3vr5}}$$

$$\mathrm{Pfbpfromfbp} = \frac{\mathrm{v1(v2v4 + v4v5 + v4vr3 + v2vr5 + vr3vr5)}}{\mathrm{v1v2v4 + v1v4v5 + v4v5vr2 + v1v4vr3 + v4vr2vr3 + v1v2vr5 + v1vr3vr5 + vr2vr3vr5}}$$

$$\mathrm{Pdhapfromfru1p} = \frac{\mathrm{v4v5(v1 + vr2)}}{\mathrm{v1v2v4 + v1v4v5 + v4v5vr2 + v1v4vr3 + v4vr2vr3 + v1v2vr5 + v1vr3vr5 + vr2vr3vr5}}$$

$$\mathrm{Pfru1pfromfru1p} = \frac{\mathrm{v4(v1v2 + v1v5 + v5vr2 + v1vr3 + vr2vr3)}}{\mathrm{v1v2v4 + v1v4v5 + v4v5vr2 + v1v4vr3 + v4vr2vr3 + v1v2vr5 + v1vr3vr5 + vr2vr3vr5}},$$

$$\mathrm{Pfbpfromfru1p} = \frac{\mathrm{v4v5vr2}}{\mathrm{v1v2v4 + v1v4v5 + v4v5vr2 + v1v4vr3 + v4vr2vr3 + v1v2vr5 + v1vr3vr5 + vr2vr3vr5}},$$

$$\mathrm{Pfr1pfromfbp} = \frac{\mathrm{v1v2vr5}}{\mathrm{v1v2v4 + v1v4v5 + v4v5vr2 + v1v4vr3 + v4vr2vr3 + v1v2vr5 + v1vr3vr5 + vr2vr3vr5}},$$

$$\mathrm{Pfbpfromdhap} = -\frac{\mathrm{(-v4vr2vr3 - vr2vr3vr5)}}{\mathrm{(v1v2v4 + v1v4v5 + v4v5vr2 + v1v4vr3 + v4vr2vr3 + v1v2vr5 + v1vr3vr5 + vr2vr3vr5)}}$$

$$\mathrm{Pfr1pfromdhap} = \frac{\mathrm{(v1vr3 + vr2vr3)vr5}}{\mathrm{v1v2v4 + v1v4v5 + v4v5vr2 + v1v4vr3 + v4vr2vr3 + v1v2vr5 + v1vr3vr5 + vr2vr3vr5}}$$

$$\mathrm{Pdhapfromdhap} = 1 - \frac{\left(\mathrm{v4v5(v1 + vr2)}\right)}{\mathrm{(v1v2v4 + v1v4v5 + v4v5vr2 + v1v4vr3 + v4vr2vr3 + v1v2vr5 + v1vr3vr5 + vr2vr3vr5)}} - \frac{\left(\mathrm{v1v2(v4 + vr5)}\right)}{\mathrm{(v1v2v4 + v1v4v5 + v4v5vr2 + v1v4vr3 + v4vr2vr3 + v1v2vr5 + v1vr3vr5 + vr2vr3vr5)}}$$

Then, with these fractions it is possible to describe the flux through each of the eight possible aldolase reactions that will be involved in label propagation.

Reaction 1: FBP → GraP + DHAP

$$J_1 = \mathrm{Pdhapfromfbp} * \mathrm{v3} = \frac{\mathrm{v3 * v1v2(v4 + vr5)}}{\mathrm{v1v2v4 + v1v4v5 + v4v5vr2 + v1v4vr3 + v4vr2vr3 + v1v2vr5 + v1vr3vr5 + vr2vr3vr5}}$$

Reaction 2: GraP + DHAP → FBP

$$J_2 = \text{Pfbpfromdhap} * \text{vr1} = -\frac{(-\text{v4vr2vr3} - \text{vr2vr3vr5})}{(\text{v1v2v4} + \text{v1v4v5} + \text{v4v5vr2} + \text{v1v4vr3} + \text{v4vr2vr3} + \text{v1v2vr5} + \text{v1vr3vr5} + \text{vr2vr3vr5})}$$

Reaction 3: Fru1P → Gra + DHAP

$$J_3 = \text{Pdhapfromfru1p} * \text{v3} = \frac{\text{v3} * \text{v4v5}(\text{v1} + \text{vr2})}{\text{v1v2v4} + \text{v1v4v5} + \text{v4v5vr2} + \text{v1v4vr3} + \text{v4vr2vr3} + \text{v1v2vr5} + \text{v1vr3vr5} + \text{vr2vr3vr5}}$$

Reaction 4: DHAP + Gra → Fru1P

$$J_4 = \text{Pfr1pfromdhap} * \text{vr4} = \frac{vr4 * (\text{v1vr3} + \text{vr2vr3})\text{vr5}}{\text{v1v2v4} + \text{v1v4v5} + \text{v4v5vr2} + \text{v1v4vr3} + \text{v4vr2vr3} + \text{v1v2vr5} + \text{v1vr3vr5} + \text{vr2vr3vr5}}$$

Reaction 5: FBP+Gra → GraP + Fru1P

$$J_4 = vr4 * \text{Pfr1pfromfbp} = \frac{\text{vr4} * \text{v1v2vr5}}{\text{v1v2v4} + \text{v1v4v5} + \text{v4v5vr2} + \text{v1v4vr3} + \text{v4vr2vr3} + \text{v1v2vr5} + \text{v1vr3vr5} + \text{vr2vr3vr5}},$$

Reaction 6: GraP+Fru1P → FBP + Gra

$$J_6 = \text{Pfbpfromfru1p} * \text{vr1} = \frac{\text{v4v5vr2}}{\text{v1v2v4} + \text{v1v4v5} + \text{v4v5vr2} + \text{v1v4vr3} + \text{v4vr2vr3} + \text{v1v2vr5} + \text{v1vr3vr5} + \text{vr2vr3vr5}},$$

Reaction 7 (Invisible reaction): FBP+GraP' ↔ GraP + FBP'

$$J_7 = \frac{v2 * v1}{v1 + vr2} - J_1 - J_4$$

Reaction 8 (Invisible reaction): Fru1P+Gra' ↔ Gra + Fru1P'

$$J_8 = \frac{v5 * v4}{v1 + vr2} - J_3 - J_6$$

Expanding these expressions with the consumption and production of metabolites derived from the King-Altman method generates the kinetic laws used in the model for each reaction, which can be used to simulate label propagation.

## References


1. Cornish-Bowden A (1995) Fundamentals of enzyme kinetics. London: Portland Press Ltd. xiii, 230 p. p.

2. Marin de Mas I, Selivanov VA, Marin S, Roca J, Orešič M, et al. (2011) Compartmentation of glycogen metabolism revealed from 13C isotopologue distributions. BMC Systems Biology 5: 175.
